# Mathematical assessment of monkeypox disease with the impact of vaccination using a fractional epidemiological modeling approach

**DOI:** 10.1038/s41598-023-40745-x

**Published:** 2023-08-20

**Authors:** Botao Liu, Samreen Farid, Saif Ullah, Mohamed Altanji, Rashid Nawaz, Shewafera Wondimagegnhu Teklu

**Affiliations:** 1 School of Mathematics and Statistics, Lanzhou University, Lanzhou, 730000 People’s Republic of China; 2 Department of Mathematics, Abdul Wali Khan University Mardan, Mardan, Pakistan; 3 Department of Mathematics, University of Peshawar, Peshawar, Khyber Pakhtunkhwa Pakistan; 4 Department of Mathematics, College of Science, King Khalid University, Abha, 61413 Saudi Arabia; 5 Department of Mathematics, Natural Science, Debre Berhan University, 445, Debre Berhan, Ethiopia

**Keywords:** Computational biology and bioinformatics, Mathematics and computing

## Abstract

This present paper aims to examine various epidemiological aspects of the monkeypox viral infection using a fractional-order mathematical model. Initially, the model is formulated using integer-order nonlinear differential equations. The imperfect vaccination is considered for human population in the model formulation. The proposed model is then reformulated using a fractional order derivative with power law to gain a deeper understanding of disease dynamics. The values of the model parameters are determined from the cumulative reported monkeypox cases in the United States during the period from May 10th to October 10th, 2022. Besides this, some of the demographic parameters are evaluated from the population of the literature. We establish sufficient conditions to ensure the existence and uniqueness of the model’s solution in the fractional case. Furthermore, the stability of the endemic equilibrium of the fractional monkeypox model is presented. The Lyapunov function approach is used to demonstrate the global stability of the model equilibria. Moreover, the fractional order model is numerically solved using an efficient numerical technique known as the fractional Adams-Bashforth-Moulton method. The numerical simulations are conducted using estimated parameters, considering various values of the fractional order of the Caputo derivative. The finding of this study reveals the impact of various model parameters and fractional order values on the dynamics and control of monkeypox.

## Introduction

Monkeypox is a viral disease caused by the monkeypox virus (MPXV), which belongs to the Orthopoxvirus genus. It primarily affects animals, particularly monkeys, squirrels, and rodents. It is a zoonotic disease that can be transmitted to humans through animals. Human-to-human transmission can also occur via close contact with infected individuals, particularly through respiratory droplets or contact with skin lesions or other body fluids. Besides these, other possible modes of transmission include contact with contaminated objects or surfaces known as environmental transmission. Although, infected cases are found in numerous countries in the world, but Africa is the main place where it is most commonly found. The first monkeypox case was identified in 1958 following the occurrence of outbreaks in a group of monkeys that were being studied. Globally, there have been a total of 88,122 reported cases of infection, with 30,555 of these cases occurring in the United States^1^. The symptoms of monkeypox virus infection in humans are comparable to smallpox signs but less severe^2,3^. Typically, the infection begins with a fever, headache, muscle pain, and tiredness^3^. The primary distinction between the symptoms of monkeypox and smallpox is that the monkeypox infection results in lymphadenopathy, characterized by the swelling of lymph nodes^3^. The incubation period for this infection usually ranges from 7 to 14 days, although it can vary between 5 and 21 days^1,3^. Symptoms of monkeypox are usually mild, and the majority of patients naturally recover within a few weeks. Those with weakened immune systems may exhibit severe symptoms^1,2,4,5^. Smallpox and human monkeypox are clinically related due to their difficult-to-distinguish characteristics^2^. Typically, monkeypox is spread to humans through contact with animal blood or bites from rodents, pets, and primates^2,3^. Gambian pouched rats (Cricetomys Gambians), dormice (*Graphiurus* sp.), and African squirrels (Heliosciurus and Funisciurus) have also been found to be infected^4^. Currently, no approved and safe treatment mechanism specifically for monkeypox virus infection. Nevertheless, some particular controlling measures can be used to mitigate the spread of monkeypox. Vaccines, immune globulin, and antiviral medications introduced for smallpox control can be employed to control the transmission. However, that the smallpox vaccine is currently unavailable as smallpox has been eradicated worldwide^6,7^.

Monkeypox has received little attention in the past, making it difficult to understand its epidemiology. Despite this, numerous mathematical models have been recently developed to analyze the dynamics of the monkeypox. A novel mathematical model based on deterministic approach for the monkeypox outbreak was developed and examined in^8^. Their findings revealed that isolating infected people from other populations reduces disease incidence. In^9^ the authors developed a system of nonlinear differential equations to analyze different modes of monkeypox transmission. The numerical simulation indicates that the immunological status of an individual affects how well they recover from an orthopoxvirus infection. To learn more about the mechanisms of transmission and various controlling methods, many epidemic models on infectious disease have been investigated^8,10–13^. In^14^ authors developed a mathematical model to better comprehend the dynamics of the monkeypox. Their results suggest the monkeypox outbreak can be eradicated through the proper implementation of non-pharmaceutical interventions. Additionally, in the study of^15^, the numerical simulation of the model demonstrated that the treatment will result in the elimination of infected individuals from human and non-human primate populations throughout the course of the investigation. Recently, in^16,17^ the authors introduce a mathematical model with environmental transmission to study the dynamics and some controlling measures of the monkeypox 2022 outbreak.

Mathematical modeling with fractional differential equations has recently attracted the attention of researchers in a wide range of scientific and professional domains, particularly in epidemiology^18–20^. The memory effect is one of the remarkable characteristics of fractional-order models that cannot be developed in classical models because of the numerous properties of fractional operators^21,22^. Many researchers have recently employed fractional differential equations to represent a variety of infectious and non-infectious diseases. One of these study subjects that has received great attention and produced insightful results is the COVID-19 infection. Mathematical models for COVID-19 based on the fractional derivatives are taken into account in^23–27^. The dynamics of monkeypox with cross-infection via a novel stochastic model was studied in^28^. A novel modeling approach based on fractal-fractional operators demonstrating monkeying dynamics with animal-to-human transmission was proposed in^29^.

This study analyzes the dynamics of monkeypox under some controlling measures using fractional-order calculus. We present a comprehensive theoretical and numerical analysis of the fractional order epidemic model. Most of the model parameters are estimated from the actual data set of the monkeypox 2022 outbreak. The description of the manuscript’s main sections is as follows: The section titled “Basic definitions” introduces some fundamental concepts of fractional derivatives. The proposed monkeypox model and parameter estimation are described in section “Formulation of the integer order monkeypox model”. The model’s qualitative analysis is presented in section “Basic analysis of the fractional monkeypox model”. A graphical interpretation of the basic reproduction number versus model parameters is presented in section “[Sec Sec12]”. Detailed numerical treatment for the fractional model is accomplished in section “Numerical results of the fractional model”. Finally, in the last section titled “Conclusion,” we summarized with a conclusion.

## Basic definitions

Fractional differential operators in mathematical modeling are well-known and it has been successful in the fields like science, engineering, and epidemiology. We give a few fundamental definitions along with some proprieties that be will used in the rest of the study^30,31^.

### Definition 1

The left and right Caputo fractional derivative operator of the function $f\in L^{\infty }(\mathbb {R})\cap C(\mathbb {R})$ is defined as follows

1 $$\begin{aligned}&_{t_0 }^c D_t^\vartheta f\left( t \right) = \left( {_{t_0 } D_t^{ - \left( {m - \vartheta } \right) } \left( {\frac{d}{{dt}}} \right) ^m f\left( t \right) } \right) = \frac{1}{{\Gamma \left( {m - \vartheta } \right) }}\int \limits _{t_0 }^t {\left( {\left( {t - l} \right) ^{m - \vartheta - 1} f^m \left( l \right) } \right) } dl, \nonumber \\&and \nonumber \\&_t^c D_T^\vartheta f\left( t \right) = \left( {_t D_T^{ - \left( {m - \vartheta } \right) } \left( { - \frac{d}{{dt}}} \right) ^m f\left( t \right) } \right) = \frac{{\left( { - 1} \right) ^m }}{{\Gamma \left( {m - \vartheta } \right) }}\int \limits _t^T {\left( {\left( {l - t} \right) ^{m - \vartheta - 1} f^m \left( l \right) } \right) } dl. \end{aligned}$$

### Definition 2

For $x\in \mathbb {R}$, the generalized Mittag-Leffler function $E_{\alpha ,\beta }(x)$ is defined by

2 $$\begin{aligned} E_{\alpha ,\beta } \left( x \right) = \sum \limits _{m = 0}^\infty {\frac{{x^m }}{{\Gamma \left( {\alpha m + \beta } \right) }}} ,\,\,\,\alpha ,\beta > 0, \end{aligned}$$

and satisfies the following property given in

3 $$\begin{aligned} E_{\alpha ,\beta } \left( x \right) = x\,E_{\alpha ,\alpha + \beta } \left( x \right) + \frac{1}{{\Gamma \left( \beta \right) }}. \end{aligned}$$

The Laplace transform of the function $t^{\beta - 1} E_{\alpha ,\beta } \left( { \pm \lambda t^\alpha } \right)$ is defined as follows

4 $$\begin{aligned} L\left[ {t^{\beta - 1} E_{\alpha ,\beta } \left( { \pm \lambda t^\alpha } \right) } \right] = \frac{{s^{\alpha - \beta } }}{{s^\alpha \mp \lambda }}. \end{aligned}$$

### Proposition 2.1

Let, $f\in L^{\infty }(\mathbb {R})\cap C(\mathbb {R})$ and $\alpha \in \mathbb {R}$, $m-1<\alpha <m$ then the following condition hold

$$\begin{aligned}{} & {} 1.\,_{t_{0}}^{c}D_{t}^{\vartheta } I^\vartheta f\left( t \right) = f\left( t \right) . \nonumber \\{} & {} 2.\,I^{\vartheta }\,_{t_{0}}^{c}D_{t}^{\vartheta } f\left( t \right) = f\left( t \right) - \sum \limits _{k = 0}^{m - 1} {\frac{{t^k }}{{k!}}f^k \left( t_{0} \right) .} \end{aligned}$$

## Formulation of the integer order monkeypox model

The total human population $N_{h}$ is divided into six different compartments i.e $S_{h}$ susceptible humans, $E_{h}$ exposed humans, $I_{h}$ infected humans, $C_{h}$ clinically ill humans, $V_{h}$ vaccinated humans and $R_{h}$ recovered individuals. Similarly, $N_{r}$ represents the overall non-human population which is further divided into four different subgroups i.e. $S_{r}$ susceptible, $E_{r}$ exposed, $I_{r}$ infected and $R_{r}$ recovered animals respectively.

The susceptible human class is initiated through recruited rate $\phi _{h}$. This population is declined by the natural mortality rate $\mu _{h}$ and by the population gaining infection after interacting with infected humans (or animals) at the contact rate $\beta _1$ or $\beta _2$ respectively. The susceptible human group is also decreased by vaccinating at the rate $\alpha _{h}$. This class is further increased by the vaccinated population after waning the induced immunity at the rate $\eta$. Thus, the dynamics of the susceptible human class are modeled via the following equation

$$\begin{aligned} \frac{dS_h}{dt} = \phi _h - \left( {\frac{{\beta _1 I_h+\beta _2 I_r}}{{N_h }}} \right) S_h -\left( {\mu _h + \alpha _h } \right) S_h + \eta V_h. \end{aligned}$$

The class of exposed individuals is increased by joining the newly infected individuals with the force of infection $\lambda _{h}=\left( {\frac{{\beta _1 I_h+\beta _2 I_r}}{{N_h }}} \right)$. Moreover, this population class is declined by the transmission rate $\beta$ and the mortality rate $\mu _{h}$. Thus, we obtained the following differential equation describing the dynamics of exposed human class

$$\begin{aligned} \frac{dE_h}{dt} =\left( {\frac{{\beta _1 I_h+\beta _2 I_r}}{{N_h }}} \right) S_h - \left( {\mu _h + \beta } \right) E_h. \end{aligned}$$

The number of infected people rises at a rate of $\beta$ after the transition from being exposed class. This class is decreased by the natural death rate $\mu _{h}$, the disease-induced death rate $\delta _{1}$, the recovered population at the $\omega _{1}$ and by the people who became clinically ill and join next class at the rate $\gamma$. Thus, the resulting dynamics can be modeled via the following equation

$$\begin{aligned} \frac{dI_h}{dt} = \beta E_h -\left( {\omega _1 + \gamma + \mu _h + \delta _1 } \right) I_h. \end{aligned}$$

The people in $I_h$ class become clinically (or critically) ill at the rate $\gamma$ and join $C_h$ class. These individuals are reduced by natural mortality rate $\mu _{h}$ and disease-induced death rate $\delta _{2}$. Further, it is decreased by joining the recovered class at the rate $\rho$. Hence, we obtained the following equation describing the dynamics of $C_h$ population

$$\begin{aligned} \frac{dC_h}{dt} = \gamma I_h -\left( {\rho + \mu _h + \delta _2 } \right) C_h. \end{aligned}$$

The susceptible individuals are vaccinated at the rate $\alpha _{h}$. The vaccinated individuals are decreased due to natural mortality rate $\mu _{h}$ and due to the waning of induced immunity at a rate $\eta$. Thus, we have

$$\begin{aligned} \frac{dV_h}{dt} =\alpha _h S_h - ( \mu _h + \eta ) V_h. \end{aligned}$$

Finally, the class of recovered humans is formed due to the transition of individuals from $I_h$ and $C_h$ at the recovery rates $\rho$ and $\omega _{1}$ respectively. Further, it is decreased due to the natural death rate $\mu _{h}$. Thus, we obtain

$$\begin{aligned} \frac{dR_h}{dt} =\rho C_h + \omega _1 I_h - \mu _h R_h. \end{aligned}$$

The recruitment rate of susceptible animals is $\phi _{r}$ and the natural death rate in all animal populations is denoted by $\mu _{r}$. The force of infection in this case is given by

$$\begin{aligned} \lambda _{r}= {\frac{{\beta _3 I_r }}{{N_r }}}, \end{aligned}$$

where $\beta _3$ is the effective contact rate causing the disease transmission rate among animals. The flow from exposed to the infected animal compartment is denoted by $\varepsilon$. The death rate induced due to infection in infected animals is $\delta _3$. The parameter $\omega _2$ shows the recovery rate of infected animals. Thus, the dynamics of monkeypox in animals are given by the following system

5 $$\begin{aligned} \frac{dS_r}{dt}= & {} \phi _r - {\left( {\frac{{\beta _3 I_r }}{{N_r }}} \right) } S_r-\mu _r S_r,\nonumber \\ \frac{dE_r}{dt}= & {} \left( {\frac{{\beta _3 I_r }}{{N_r }}} \right) S_r -\left( {\varepsilon + \mu _r } \right) E_r,\nonumber \\ \frac{dI_r}{dt}= & {} \varepsilon E_r - \left( {\omega _2 + \mu _h + \delta _3 } \right) I_r,\nonumber \\ \frac{dR_r}{dt}= & {} \omega _2 I_r - \mu _r R_r. \end{aligned}$$

### Fractional monkeypox model in the Caputo sense

This section presents the extension of the integer order monkeypox model to the fractional case. The well-known Caputo derivative having order $0<\vartheta \le 1$ is utilized to formulate the generalized fractional epidemic model. Fractional epidemic models used fractional derivatives to capture more complex and non-local effects in the disease transmission dynamics. Such epidemic models have a greater degree of freedom and offer insights into the behavior and control of infectious diseases for a particular set of data^22^. The proposed model for transmission of monkeypox model is described by the following system

6 $$\begin{aligned} ^c D_t^\vartheta S_h= & {} \phi _h - \lambda _h S_h - \texttt {k}_1 S_h + \eta V_h, \nonumber \\ ^c D_t^\vartheta E_h= & {} \lambda _h S_h - \texttt {k}_2 E_h, \nonumber \\ ^c D_t^\vartheta I_h= & {} \beta E_h - \texttt {k}_3 I_h, \nonumber \\ ^c D_t^\vartheta C_h= & {} \gamma I_h - \texttt {k}_4 C_h, \nonumber \\ ^c D_t^\vartheta V_h= & {} \alpha _h S_h - \texttt {k}_5 V_h, \nonumber \\ ^c D_t^\vartheta R_h= & {} \rho C_h + \omega _1 I_h - \mu _h R_h, \nonumber \\ ^c D_t^\vartheta S_r= & {} \phi _r - \left( {\lambda _r + \mu _r } \right) S_r, \nonumber \\ ^c D_t^\vartheta E_r= & {} \lambda _r S_r - \texttt {k}_6 E_r, \nonumber \\ ^c D_t^\vartheta I_r= & {} \varepsilon E_r - \texttt {k}_7 I_r, \nonumber \\ ^c D_t^\vartheta R_r= & {} \omega _2 I_r - \mu _r R_r, \end{aligned}$$

where,

$$\begin{aligned} \,\texttt {k}_1&= \left( {\mu _h + \alpha _h } \right) ,\,\,\texttt {k}_2 = \left( {\mu _h + \beta } \right) ,\,\,\texttt {k}_3 = \left( {\omega _1 + \gamma + \mu _h + \delta _1 } \right) ,\,\texttt {k}_4 = \left( {\delta + \mu _h + \delta _2 } \right) ,\texttt {k}_5 = \left( {\mu _h + \eta } \right) ,\,\\ \texttt {k}_6&= \left( {\varepsilon + \mu _r } \right) \, \,\texttt {k}_7 = \left( {\omega _2 + \mu _h + \delta _3 } \right) . \end{aligned}$$

Subject to non-negative initial conditions

7 $$\begin{aligned} S_h \left( 0 \right)= & {} \mathord{\buildrel{\lower3pt\hbox{$\scriptscriptstyle\smile$}} \over S} _{h_0},E_h \left( 0 \right) = \mathord{\buildrel{\lower3pt\hbox{$\scriptscriptstyle\smile$}} \over E} _{h_0 },I_h \left( 0 \right) = \mathord{\buildrel{\lower3pt\hbox{$\scriptscriptstyle\smile$}} \over I} _{h_0 },C_h \left( 0 \right) = \mathord{\buildrel{\lower3pt\hbox{$\scriptscriptstyle\smile$}} \over C} _{h_0 },V_h \left( 0 \right) = \mathord{\buildrel{\lower3pt\hbox{$\scriptscriptstyle\smile$}} \over V} _{h_0 },R_h \left( 0 \right) = \mathord{\buildrel{\lower3pt\hbox{$\scriptscriptstyle\smile$}} \over R} _{h_0 }, \nonumber \\ S_r \left( 0 \right)= & {} \mathord{\buildrel{\lower3pt\hbox{$\scriptscriptstyle\smile$}} \over S} _{r_0 },E_r \left( 0 \right) = \mathord{\buildrel{\lower3pt\hbox{$\scriptscriptstyle\smile$}} \over E} _{r_0 },I_r \left( 0 \right) = \mathord{\buildrel{\lower3pt\hbox{$\scriptscriptstyle\smile$}} \over I} _{r_0 } and\,R_r \left( 0 \right) = \mathord{\buildrel{\lower3pt\hbox{$\scriptscriptstyle\smile$}} \over R} _{r_0 }. \end{aligned}$$

### Parameter estimation and data fitting

The objective of the present section is to accomplish the parameter estimation of the model. The estimation procedure is performed in two ways. Most of the model parameters are estimated from the real statistics of infected cases reported from 10 May to the end of October 2022 in the recent outbreak in the USA^1,3^. While some of the demographic parameters (the natural death and birth rate) are estimated from the USA population^32^. The estimation of parameters from the real disease data is significant for carrying out the numerical simulations more realistically. The estimation process of the proposed model (6) is conducted using the well-known nonlinear least square technique.

This procedure involves the following main steps:

1. Define an objective function for quantifying the difference between the predicted and the actual data. This function is mathematically expressed as the sum of squared residuals (SSR) between the model predictions and the corresponding actual data points.
2. Define or set the model parameters’ initial values that need to be estimated. These values can be based on prior knowledge or initial guesses.
3. Utilizing the initial parameter values set in step 2, the epidemic model is simulated in order to generate model predictions.
4. Using a ’lsqcurvefit’ optimization technique, for the minimization of objective function.
5. Set a convergence criterion to stop the iterative estimation scheme. This criterion is based on a maximum number of iterations, a minimal change in parameter estimates, or attaining a predefined threshold for the corresponding objective function.
6. If the estimated parameter values do not adequately fit the real data curve or do not meet the convergence criterion, reset the initial parameter estimates and re-execute steps 3–5 until a reasonable agreement between the model simulation and the actual data is obtained.

**Figure 1:**
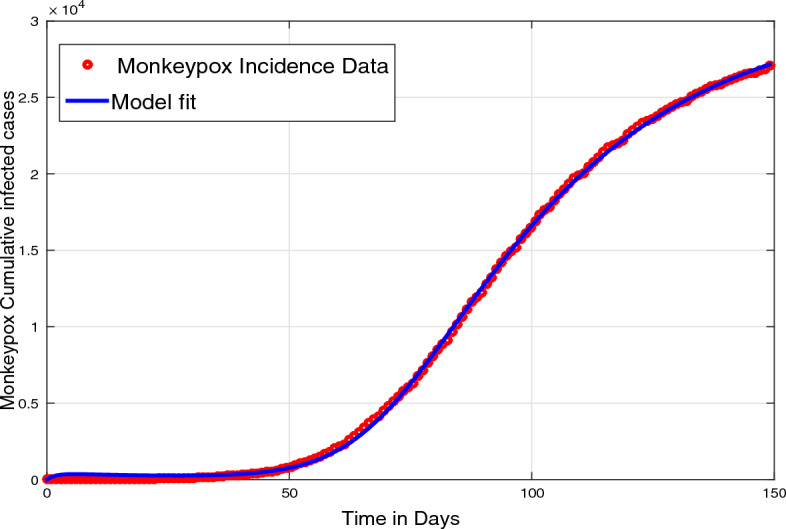
Model predicted curve (solid line) to the observed data for the case when $\vartheta =1$.

By iteratively minimizing the objective function via a standard nonlinear least squares technique, the parameter estimates are refined, which leads to improved agreement between the model-predicted and observed data. The fitted and estimated values of the monkeypox epidemic model’s parameters are listed in Table 1, while Fig. 1 illustrates the model’s good fit to the observed data.

**Table 1:** Fitted and estimated values of the model parameters.

Parameter	Description	Value in days	References
$\phi _{h}$	Birth rate of humans	11731.91	^32^
$\phi _{r}$	Birth rate of animals or rodents	0.016	^8^
$\eta$	Waning induced immunity	0.2010	Fitted
$\rho$	Recovery rate of $C_{h}$	0.0843	Fitted
$\beta$	Transition rate of $E_{h}$ to $I_{h}$	0.0486	Fitted
$\gamma$	Moving of $I_{h}$ to $C_{h}$	0.1119	Fitted
$\beta _3$	Contact rate of $S_{r}$ and $I_{r}$	0.2461	Fitted
$\beta _{1}$	Contact rate of $S_{h}$ and $I_{h}$	0.5701	Fitted
$\beta _{2}$	Contact rate between $I_{r}$ and $S_{h}$	0.2508	Fitted
$\alpha _{h}$	Vaccinated against monkeypox	0.2393	Fitted
$\mu _{h}$	Natural death rate of humans	1/(79*365)	^32^
$\delta _{1}$	Disease induced mortality rate of infectious human	0.0011	Fitted
$\delta _{2}$	Disease-induced rate of $C_{h}$	1.0091e−04	Fitted
$\delta _{3}$	Disease induced death rate of infected animals	1.5303e−05	Fitted
$\omega _{1}$	Permanent immunity due to treatment	0.4670	Fitted
$\mu _{r}$	Natural death rate of animals	0.000016	^8^
$\omega _{2}$	Recovery rate due to immunity	0–1	Assumed
$\epsilon$	Progression from being $E_{r}$ to $I_{r}$	0.1053	Fitted

## Basic analysis of the fractional monkeypox model

In this section, we address some of the basic and necessary mathematical analysis of the fractional monkeypox epidemic model (6). We proceed as follows

### Positivity and boundedness

For the positive initial values $\mathop {\lim }\limits _{t \rightarrow \infty } \sup N_h \left( t \right) \le \frac{{\phi _h }}{{\mu _h }}\,\,\,\,and\,\,\mathop {\lim }\limits _{t \rightarrow \infty } \sup N_r \left( t \right) \le \frac{{\phi _r }}{{\mu _r }}\,$. Also, if $N_{h_0 } \left( t \right) \le \frac{{\phi _h }}{{\mu _h }}\,\,\,\,and\,\,N_{r_0 } \left( t \right) \le \frac{{\phi _r }}{{\mu _r }}\,$ then the feasible domain for the given system is given by

$$\begin{aligned} \Omega _h&= \left\{ {\left( {S_h ,E_h ,I_h ,C_h ,V_h ,R_h } \right) \subset \mathbb {R}_ + ^6 :S_h + E_h + I_h + C_h + V_h + R_h \le \frac{{\phi _h }}{{\mu _h }}} \right\} , \\ \Omega _r&= \left\{ {\left( {S_r ,E_r ,I_r ,R_r } \right) \subset \mathbb {R}_ + ^4 :S_r + E_r + I_r + R_r \le \frac{{\phi _r }}{{\mu _r }}} \right\} , \\ \end{aligned}$$

such that $\Omega = \Omega _h \times \Omega _r \subset \mathbb {R}_ + ^6 \times \mathbb {R}_ + ^4$ and we have $\Omega$ is positively invariant.

Let $$\mathord{\buildrel{\lower3pt\hbox{$\scriptscriptstyle\smile$}} \over S} _{h_0 } \mathord{\buildrel{\lower3pt\hbox{$\scriptscriptstyle\smile$}} \over E} _{h_0 } ,\mathord{\buildrel{\lower3pt\hbox{$\scriptscriptstyle\smile$}} \over I} _{h_0 } ,\mathord{\buildrel{\lower3pt\hbox{$\scriptscriptstyle\smile$}} \over C} _{h_0 } ,\mathord{\buildrel{\lower3pt\hbox{$\scriptscriptstyle\smile$}} \over V} _{h_0 } ,\mathord{\buildrel{\lower3pt\hbox{$\scriptscriptstyle\smile$}} \over R} _{h_0 } ,$$ $$\mathord{\buildrel{\lower3pt\hbox{$\scriptscriptstyle\smile$}} \over S} _{r_0 } ,\mathord{\buildrel{\lower3pt\hbox{$\scriptscriptstyle\smile$}} \over E} _{r_0 } ,\mathord{\buildrel{\lower3pt\hbox{$\scriptscriptstyle\smile$}} \over I} _{r_0 } $$ and $$ \mathord{\buildrel{\lower3pt\hbox{$\scriptscriptstyle\smile$}} \over R} _{r_0 } $$ be positive, then all solution are will be positive for $$t>0$$. Considering the first equation in (6) we have

$$\begin{aligned} ^c D_t^\vartheta S_h = \phi _h - \left( {\lambda _h + \texttt {k}_1 } \right) S_h + \eta V_h, \end{aligned}$$

then

$$\begin{aligned} ^c D_t^\vartheta S_h + \left( {\lambda _h + \texttt {k}_1 } \right) S_h = \phi _h + \eta V_h. \end{aligned}$$

Since $\phi _h + \eta \,V_h \ge 0$ therefore, it implies

$$\begin{aligned} ^c D_t^\vartheta S_h + \left( {\lambda _h + \texttt {k}_1 } \right) S_h \ge 0. \end{aligned}$$

By using the Laplace transform we have

$$\begin{aligned}&L\left[ {^c D_t^\vartheta S_h } \right] + L\left[ {\left( {\lambda _h + \texttt {k}_1 } \right) S_h } \right] \ge 0, \\&s^\vartheta S_h \left( s \right) - s^{\vartheta - 1} S_h \left( 0 \right) + \left( {\lambda _h + \texttt {k}_1 } \right) S_h \left( s \right) \ge 0, \\&S_h \left( s \right) \ge \frac{{s^{\vartheta - 1} }}{{\left( {s^\vartheta + \left( {\lambda _h + \texttt {k}_1 } \right) } \right) }}S_{h_0 }. \end{aligned}$$

Applying inverse Laplace transform we have

$$\begin{aligned} S_h \left( t \right) \ge E_{\vartheta ,1} \left( { - \left( {\lambda _h + \texttt {k}_1 } \right) t^\vartheta } \right) S_{h_0 }. \end{aligned}$$

Since, quantities on right hand side are positive so we have $S_{h}\ge 0$ for $t\ge 0$. In similar approach, we have $E_h ,I_h ,C_h ,V_h ,R_h ,S_r ,E_r ,I_r \,and\,R_r \ge 0\,\,\,\forall t > 0$ corresponding to any non-negative initial values. Thus, the solution remain in $\mathbb {R}_ + ^6 \times \mathbb {R}_ + ^4$ for all $t>0$ with non-negative initial values.

Next to we prove the boundedness of the fractional model solution. The total human individuals is given by

$$\begin{aligned} N_h \left( t \right) = S_h \left( t \right) + E_h \left( t \right) + I_h \left( t \right) + C_h \left( t \right) + V_h \left( t \right) + R_h \left( t \right) , \end{aligned}$$

such that

$$\begin{aligned} ^c D_t^\vartheta N_h \left( t \right)&= ^c D_t^\vartheta S_h \left( t \right) + ^c D_t^\vartheta E_h \left( t \right) + ^c D_t^\vartheta I_h \left( t \right) + ^c D_t^\vartheta C_h \left( t \right) + ^c D_t^\vartheta V_h \left( t \right) + ^c D_t^\vartheta R_h \left( t \right) , \\ ^c D_t^\vartheta N_h \left( t \right)&= \phi _h - \left( {\delta _1 I_h + \delta _2 C_h } \right) - \mu _h N_h \left( t \right) \le \phi _h - \mu _h N\left( t \right) , \\ ^c D_t^\vartheta N_h \left( t \right)&\le \phi _h - \mu _h N_h \left( t \right) . \end{aligned}$$

Taking Laplace transform on both sides we have

$$\begin{aligned}&L\left[ {^c D_t^\vartheta N_h \left( t \right) } \right] \le L\left[ {\phi _h - \mu _h N_h \left( t \right) } \right] , \\&s^\vartheta N_h \left( s \right) - s^{\vartheta - 1} N_h \left( 0 \right) + \mu _h N_h \left( s \right) \le \frac{{\phi _h }}{s}, \\&N_h \left( s \right) \le \frac{{s^{\vartheta - 1} }}{{\left( {s^\vartheta + \mu _h } \right) }}N_h \left( 0 \right) + \frac{{\phi _h }}{{s\left( {s^\vartheta + \mu _h } \right) .}} \end{aligned}$$

By applying inverse Laplace transform we have

8 $$\begin{aligned} N_h \left( t \right) \le E_{\vartheta ,1} \left( { - \mu _h t^\vartheta } \right) N_h \left( 0 \right) + \phi _h E_{\vartheta ,\vartheta + 1} \left( { - \mu _h t^\vartheta } \right) . \end{aligned}$$

By taking limit $t\rightarrow \infty$ we have $N_{h}(t) \le \frac{\phi _{h}}{\mu _{h}}$ implies that $\mathop {\lim }\limits _{t \rightarrow \infty } \sup N_h \left( t \right) = \frac{{\phi _h }}{{\mu _h }}.$ If $N_{h_0 } \le \frac{{\phi _h }}{{\mu _h }}$ then $N_h \left( t \right) \le \frac{{\phi _h }}{{\mu _h }}$ which implies $N_{h}(t)$ is bounded. In similar way, we can prove that $N_{r}(t)$ is bounded. Therefore, this established the notion of the set $\Omega$ as required. So, we conclude that the region is epidemiological feasible and well posed in $\Omega$.

### Existence and uniqueness

Let *T* be a positive real number and consider $J=[0,T]$. We denote the set of all continuous function defined on *J* by $C^{0}_{e}(J)$ with norm as $\left\| X \right\| = \sup \left\{ {\left| {X\left( t \right) } \right| ,t \in J} \right\} .$ Consider the system (6), (7) as following initial value problem (I.V.P.)

9 $$\begin{aligned} \left\{ \begin{array}{l} ^c D_t^\vartheta X\left( t \right) = F\left( {t,X\left( t \right) } \right) ,0< t< T < \infty , \\ X\left( 0 \right) = X_0 , \\ \end{array} \right. \end{aligned}$$

where $X(t)=(S_{h}(t),E_{h}(t),I_{h}(t),C_{h}(t),V_{h}(t),R_{h}(t),S_{r}(t),E_{r}(t),I_{r}(t),R_{r}(t))$ represents the compartments and *F* is continuous function defined as follows

10 $$\begin{aligned} F\left( {t,X\left( t \right) } \right) = \left( {\begin{array}{*{20}l} {F_1 \left( {t,S_h\left( t \right) } \right) } \\ {F_2 \left( {t,E_h\left( t \right) } \right) } \\ {F_3 \left( {t,I_h\left( t \right) } \right) } \\ {F_4 \left( {t,C_h\left( t \right) } \right) } \\ {F_5 \left( {t,V_h\left( t \right) } \right) } \\ {F_6 \left( {t,R_h\left( t \right) } \right) } \\ {F_7 \left( {t,S_r\left( t \right) } \right) } \\ {F_8 \left( {t,E_r\left( t \right) } \right) } \\ {F_9 \left( {t,I_r\left( t \right) } \right) } \\ {F_{10} \left( {t,R_r\left( t \right) } \right) } \\ \end{array}} \right) = \left( {\begin{array}{*{20}l} {\phi _h - \left( {\frac{{\beta _1 I_h +\beta _2 I_r }}{{N_h }} + \mu _h } \right) S_h - \alpha _h S_h + \eta \,V_h } \\ {\left( {\frac{{\beta _1 I_h+\beta _2 I_r }}{{N_h }}} \right) S_h - \left( {\mu _h + \beta } \right) E_h } \\ {\beta E_h - \left( {\omega _1 + \gamma + \mu _h + \delta _1 } \right) I_h } \\ {\gamma I_h - \left( {\rho + \mu _h + \delta _2 } \right) C_h } \\ {\alpha _h S_h - \mu _h V_h - \eta \,V_h } \\ {\rho C_h + \omega _1 I_h - \mu _h R_h } \\ {\phi _r - \left( {\frac{{\beta _3 I_r }}{{N_r }} + \mu _r } \right) S_r } \\ {\left( {\frac{{\beta _3 I_r }}{{N_r }}} \right) S_r - \left( {\varepsilon + \mu _r } \right) E_r } \\ {\varepsilon E_r - \left( {\mu _r + \delta _3 + \omega _2 } \right) I_r } \\ {\omega _2 I_r - \mu _r R_r } \\ \end{array}} \right) . \end{aligned}$$

Using property (2.1) in proposition we have

$$\begin{aligned} S_h \left( t \right)&= \mathord{\buildrel{\lower3pt\hbox{$\scriptscriptstyle\smile$}} \over S} _{h_0 } + I_t^\vartheta \left( {\phi _h - \left( {\frac{{\beta _1 I_h+\beta _2 I_r }}{{N_h }} + \mu _h } \right) S_h - \alpha _h S_h + \eta \,V_h } \right) , \\\\ E_h \left( t \right)&= \mathord{\buildrel{\lower3pt\hbox{$\scriptscriptstyle\smile$}} \over E} _{h_0 } + I_t^\vartheta \left( {\left( {\frac{{\beta _1 I_h+\beta _2 I_r }}{{N_h }}} \right) S_h - \left( {\mu _h + \beta } \right) E_h } \right) , \\\\ I_h \left( t \right)&= \mathord{\buildrel{\lower3pt\hbox{$\scriptscriptstyle\smile$}} \over I} _{h_0 } + I_t^\vartheta \left( {\beta E_h - \left( {\omega _1 + \gamma + \mu _h + \delta _1 } \right) I_h } \right) , \\\\ C_h \left( t \right)&=\mathord{\buildrel{\lower3pt\hbox{$\scriptscriptstyle\smile$}} \over C} _{h_0 } + I_t^\vartheta \left( {\gamma I_h - \left( {\rho + \mu _h + \delta _2 } \right) C_h } \right) , \\\\ V_h \left( t \right)&=\mathord{\buildrel{\lower3pt\hbox{$\scriptscriptstyle\smile$}} \over V} _{h_0 } + I_t^\vartheta \left( {\alpha _h S_h - \mu _h V_h - \eta \,V_h } \right) , \\\\ R_h \left( t \right)&=\mathord{\buildrel{\lower3pt\hbox{$\scriptscriptstyle\smile$}} \over R} _{h_0 } + I_t^\vartheta \left( {\rho C_h + \omega _1 I_h - \mu _h R_h } \right) , \\\\ S_r \left( t \right)&= \mathord{\buildrel{\lower3pt\hbox{$\scriptscriptstyle\smile$}} \over S} _{r_0 } + I_t^\vartheta \left( {\phi _r - \left( {\frac{{\beta _3 I_r }}{{N_r }} + \mu _r } \right) S_r } \right) , \\\\ E_r \left( t \right)&=\mathord{\buildrel{\lower3pt\hbox{$\scriptscriptstyle\smile$}} \over E} _{r_0 } + I_t^\vartheta \left( {\left( {\frac{{\beta _3 I_r }}{{N_r }}} \right) S_r - \left( {\varepsilon + \mu _r } \right) E_r } \right) , \\\\ I_r \left( t \right)&= \mathord{\buildrel{\lower3pt\hbox{$\scriptscriptstyle\smile$}} \over I} _{r_0 } + I_t^\vartheta \left( {\varepsilon E_r - \left( {\mu _r + \delta _3 + \omega _2 } \right) I_r } \right) , \\\\ R_r \left( t \right)&=\mathord{\buildrel{\lower3pt\hbox{$\scriptscriptstyle\smile$}} \over R} _{r_0 } + I_t^\vartheta \left( {\omega _2 I_r - \mu _r R_r } \right) . \\ \end{aligned}$$

The Picard iterations lead as follows

$$\begin{aligned} {S}_{h_n} \left( t \right)&=\mathord{\buildrel{\lower3pt\hbox{$\scriptscriptstyle\smile$}} \over S} _{h_0 } + \frac{1}{{\Gamma \left( \vartheta \right) }}\int \limits _0^t {\left( {t - l} \right) ^{\vartheta - 1} F_1 \left( {l,{S}_{h_{n-1}} \left( l \right) } \right) }dl, \\\\ {E}_{h_n} \left( t \right)&= \mathord{\buildrel{\lower3pt\hbox{$\scriptscriptstyle\smile$}} \over E} _{h_0 } + \frac{1}{{\Gamma \left( \vartheta \right) }}\int \limits _0^t {\left( {t - l} \right) ^{\vartheta - 1} F_2 \left( {l,{E}_{h_{n-1}} \left( l \right) } \right) }dl, \\\\ {I}_{h_n} \left( t \right)&= \mathord{\buildrel{\lower3pt\hbox{$\scriptscriptstyle\smile$}} \over I} _{h_0 } + \frac{1}{{\Gamma \left( \vartheta \right) }}\int \limits _0^t {\left( {t - l} \right) ^{\vartheta - 1} F_3 \left( {l,{I}_{h_{n-1}} \left( l \right) } \right) }dl, \\\\ {C}_{h_n} \left( t \right)&= \mathord{\buildrel{\lower3pt\hbox{$\scriptscriptstyle\smile$}} \over C} _{h_0 } + \frac{1}{{\Gamma \left( \vartheta \right) }}\int \limits _0^t {\left( {t - l} \right) ^{\vartheta - 1} F_4 \left( {l,{C}_{h_{n-1}} \left( l \right) } \right) }dl, \\\\ {V}_{h_n} \left( t \right)&= \mathord{\buildrel{\lower3pt\hbox{$\scriptscriptstyle\smile$}} \over V} _{h_0 } + \frac{1}{{\Gamma \left( \vartheta \right) }}\int \limits _0^t {\left( {t - l} \right) ^{\vartheta - 1} F_5 \left( {l,{V}_{h_{n-1}} \left( l \right) } \right) }dl, \\\\ {R}_{h_n}\left( t \right)&= \mathord{\buildrel{\lower3pt\hbox{$\scriptscriptstyle\smile$}} \over R} _{h_0 } + \frac{1}{{\Gamma \left( \vartheta \right) }}\int \limits _0^t {\left( {t - l} \right) ^{\vartheta - 1} F_6 \left( {l,{R}_{h_{n-1}} \left( l \right) } \right) }dl, \\\\ {S}_{r_n} \left( t \right)&=\mathord{\buildrel{\lower3pt\hbox{$\scriptscriptstyle\smile$}} \over S} _{r_0 } + \frac{1}{{\Gamma \left( \vartheta \right) }}\int \limits _0^t {\left( {t - l} \right) ^{\vartheta - 1} F_7 \left( {l,{S}_{r_{n-1}} \left( l \right) } \right) }dl, \\\\ {E}_{r_n} \left( t \right)&= \mathord{\buildrel{\lower3pt\hbox{$\scriptscriptstyle\smile$}} \over E} _{r_0 } + \frac{1}{{\Gamma \left( \vartheta \right) }}\int \limits _0^t {\left( {t - l} \right) ^{\vartheta - 1} F_8 \left( {l,{E}_{r_{n-1}} \left( l \right) } \right) }dl, \\\\ {I}_{r_n} \left( t \right)&= \mathord{\buildrel{\lower3pt\hbox{$\scriptscriptstyle\smile$}} \over I} _{r_0 } + \frac{1}{{\Gamma \left( \vartheta \right) }}\int \limits _0^t {\left( {t - l} \right) ^{\vartheta - 1} F_9 \left( {l,{I}_{r_{n-1}}\left( l \right) } \right) }dl, \\\\ {R}_{r_n} \left( t \right)&= \mathord{\buildrel{\lower3pt\hbox{$\scriptscriptstyle\smile$}} \over R} _{r_0 } + \frac{1}{{\Gamma \left( \vartheta \right) }}\int \limits _0^t {\left( {t - l} \right) ^{\vartheta - 1} F_{10} \left( {l,{R}_{r_{n-1}} \left( l \right) } \right) }dl. \\ \end{aligned}$$

Final transformation of the I.V.P. (9) can be written as follows

11 $$\begin{aligned} X\left( t \right) = X\left( 0 \right) + \frac{1}{{\Gamma \left( \vartheta \right) }}\int \limits _0^t {\left( {t - l} \right) ^{\vartheta - 1} F\left( {l,X\left( l \right) } \right) }dl . \end{aligned}$$

#### Lemma 1

The vector *F*(*t*, *X*(*t*)) described in (10) fulfills the well-known Lipschitz condition in the variable *X* on a set $[0,T] \times \mathbb {R}_{+}^{10}$ with Lipschitz constant

$$\begin{aligned} \psi = \max \left( \begin{array}{l} \left( {\beta _1^* + \beta _2^* + \mu _h } \right) ,\left( {\mu _h + \beta } \right) ,\left( {\mu _h + \delta _1 + \gamma + \omega _1 } \right) ,\left( {\mu _h + \rho + \delta _2 } \right) \\ ,\left( {\mu _h + \eta } \right) ,\mu _h ,\left( {\mu _r + \beta _3 } \right) ,\left( {\mu _r + \varepsilon } \right) ,\left( {\mu _r + \omega _2 + \delta _3 } \right) ,\mu _r \\ \end{array} \right) . \\ \end{aligned}$$

#### Proof

$$\begin{aligned} \begin{array}{l} \left\| {F_1 \left( {t,S_h } \right) - F_1 \left( {t,S_{h_1 } } \right) } \right\| = \left\| \begin{array}{l} \left( {\phi _h - \left( {\frac{{\beta _1 I_h+\beta _2 I_r }}{{N_h }} + \mu _h } \right) S_h - \alpha _h S_h + \eta \,V_h } \right) - \\ \left( {\phi _h - \left( {\frac{{\beta _1 I_h+\beta _2 I_r }}{{N_h }} + \mu _h } \right) S_{h_1 } - \alpha _h S_{h_1 } + \eta \,V_h } \right) \\ \end{array} \right\| \\ \,\,\,\,\,\,\,\,\,\,\,\,\,\,\,\,\,\,\,\,\,\,\,\,\,\,\,\,\,\,\,\,\,\,\,\,\,\,\,\,\,\,\,\,\,\,\,\,\,\,\,\,\,\,\,\,\,\,\,\,\,\,\,\,\,\,\,\,\,\,\ = \left\| { - \left( {\left( {\frac{{\beta _1 I_h+\beta _2 I_r }}{{N_h }}} \right) \left( {S_h - S_{h_1 } } \right) + \mu _h \left( {S_h - S_{h_1 } } \right) } \right) } \right\| \\ \,\,\,\,\,\,\,\,\,\,\,\,\,\,\,\,\,\,\,\,\,\,\,\,\,\,\,\,\,\,\,\,\,\,\,\,\,\,\,\,\,\,\,\,\,\,\,\,\,\,\,\,\,\,\,\,\,\,\,\,\,\,\,\ \le \left( {\beta _1^* + \beta _2^* } \right) \left\| {S_h - S_{h_1 } } \right\| + \mu _h \left\| {S_h - S_{h_1 } } \right\| \\ \,\,\,\,\,\,\,\,\,\,\,\,\,\,\,\,\,\,\,\,\,\,\,\,\,\,\,\,\,\,\,\,\,\,\,\,\,\,\,\,\,\,\,\,\,\,\,\,\,\,\,\,\,\,\,\,\,\,\,\,\,\ \le \,\left( {\beta _1^* + \beta _2^* + \mu _h } \right) \,\left\| {S_h - S_{h_1 } } \right\| . \\ \end{array} \end{aligned}$$

Similarly,

$$\begin{aligned} \left\| {F_2 \left( {t,E_h } \right) - F_2 \left( {t,E_{h_1 } } \right) } \right\|&\le \left( {\mu _h + \beta } \right) \left\| {E_h - E_{h_1 } } \right\| , \\\\ \left\| {F_3 \left( {t,I_h} \right) - F_3 \left( {t,I_{h_1 } } \right) } \right\|&\le \left( {\mu _h + \delta _1 + \gamma + \omega _1 } \right) \left\| {I_h - I_{h_1 } } \right\| , \\\\ \left\| {F_4 \left( {t,C_h } \right) - F_4 \left( {t,C_{h_1 } } \right) } \right\|&\le \left( {\mu _h + \rho + \delta _2 } \right) \left\| {C_h - C_{h_1 } } \right\| , \\\\ \left\| {F_5 \left( {t,V_h } \right) - F_5 \left( {t,V_{h_1 } } \right) } \right\|&\le \left( {\mu _h + \eta } \right) \left\| {V_h - V_{h_1 } } \right\| , \\\\ \left\| {F_6 \left( {t,R_h} \right) - F_6 \left( {t,R_{h_1 }} \right) } \right\|&\le \mu _h \left\| {R_h - R_{h_1 } } \right\| , \\\\ \left\| {F_7 \left( {t,S_r} \right) - F_7 \left( {t,S_{r_1 } } \right) } \right\|&\le \left( {\mu _r + \beta _3 } \right) \left\| {S_r - S_{r_1 } } \right\| , \\\\ \left\| {F_8 \left( {t,E_r } \right) - F_8 \left( {t,E_{r_1 } } \right) } \right\|&\le \left( {\mu _r + \varepsilon } \right) \left\| {E_r - E_{r_1 } } \right\| , \\\\ \left\| {F_9 \left( {t,I_r } \right) - F_9 \left( {t,I_{r_1 } } \right) } \right\|&\le \left( {\mu _r + \omega _2 + \delta _3 } \right) \left\| {I_r - I_{r_1 } } \right\| , \\\\ \left\| {F_{10} \left( {t,R_r } \right) - F_{10} \left( {t,R_{r_1 } } \right) } \right\|&\le \mu _r \left\| {R_r - R_{r_1 } } \right\| . \\ \end{aligned}$$

Combining all these we have

12 $$\begin{aligned} \begin{array}{l} \left\| {F\left( {t,X_1 \left( t \right) } \right) - F\left( {t,X_2 \left( t \right) } \right) } \right\| \le \psi \left\| {X_1 - X_2 } \right\| , \\ \psi = \max \left( \begin{array}{l} \left( {\beta _1^* + \beta _2^* + \mu _h } \right) ,\left( {\mu _h + \beta } \right) ,\left( {\mu _h + \delta _1 + \gamma + \omega _1 } \right) ,\left( {\mu _h + \rho + \delta _2 } \right) \\ ,\left( {\mu _h + \eta } \right) ,\mu _h ,\left( {\mu _r + \beta _3 } \right) ,\left( {\mu _r + \varepsilon } \right) ,\left( {\mu _r + \omega _2 + \delta _3 } \right) ,\mu _r \\ \end{array} \right) . \\ \end{array} \end{aligned}$$

$$\square $$

#### Lemma 2

Suppose we have (12) then the I.V.P. (6), (7) have unique solution $X\left( t \right) \in C_e^0 \left( J \right) .$

#### Proof

For the required result, the fixed point theory and Picard-Lindelöf is used. Solution of the (6), (7) is considered as $X(t)=W(X(t)),$ where *W* is the Picard operator defined as $W:C_e^0 \left( {J,\mathbb {R}_ + ^{10} } \right) \rightarrow C_e^0 \left( {J,\mathbb {R}_ + ^{10} } \right)$

$$\begin{aligned} W\left( {X\left( t \right) } \right) = X\left( 0 \right) + \frac{1}{{\Gamma \left( \vartheta \right) }}\int \limits _0^t {\left( {t - l} \right) ^{\vartheta - 1} F\left( {l,X\left( l \right) } \right) dl.} \end{aligned}$$

Further, it leads to

$$\begin{aligned} \begin{array}{l} \left\| {W\left( {X_1 \left( t \right) } \right) - W\left( {X_2 \left( t \right) } \right) } \right\| = \left\| {\frac{1}{{\Gamma \left( \vartheta \right) }}\int \limits _0^t {\left( {t - l} \right) ^{\vartheta - 1} \left[ {F\left( {l,X_1 \left( l \right) } \right) - F\left( {l,X_2 \left( l \right) } \right) } \right] dl} } \right\| \\ \,\,\,\,\,\,\,\,\,\,\,\,\,\,\,\,\,\,\,\,\,\,\,\,\,\,\,\,\,\,\,\,\,\,\,\,\,\,\,\,\,\,\,\,\,\,\,\,\,\,\,\, \le \frac{1}{{\Gamma \left( \vartheta \right) }}\int \limits _0^t {\left( {t - l} \right) ^{\vartheta - 1} \left\| {F\left( {l,X_1 \left( l \right) } \right) - F\left( {l,X_2 \left( l \right) } \right) } \right\| } dl \\ \,\,\,\,\,\,\,\,\,\,\,\,\,\,\,\,\,\,\,\,\,\,\,\,\,\,\,\,\,\,\,\,\,\,\,\,\,\,\,\,\,\,\,\,\,\,\,\,\,\, \le \frac{\psi }{{\Gamma \left( \vartheta \right) }}\int \limits _0^t {\left( {t - l} \right) ^{\vartheta - 1} } \left\| {X_1 - X_2 } \right\| dl \\ \,\,\,\,\,\,\,\,\,\,\,\,\,\,\,\,\,\,\,\,\,\,\,\,\,\,\,\,\,\,\,\,\,\,\,\,\,\,\,\,\,\,\,\,\,\,\,\,\, \le \frac{\psi }{{\Gamma \left( {\vartheta + 1} \right) }}W. \\ \end{array} \end{aligned}$$

If $\frac{\psi }{{\Gamma \left( {\vartheta + 1} \right) }}W <1$, then *W* gives a contraction and therefore, the problem (6), (7) has a unique solution. $\square$

### Model equilibria and the basic reproduction number $\mathcal {R}_{0}$

The model disease free equilibrium (DFE) is given by

13 $$\begin{aligned} D_0 = \left( {S_h^0 ,E_h^0 ,I_h^0,C_h^0 ,V_h^0 ,R_h^0 ,S_r^0 ,E_r^0 ,I_r^0 ,R_r^0 } \right) = \left( {\frac{{\texttt {k}_5 \phi _h }}{{\texttt {k}_1 \texttt {k}_5 - \eta \alpha _h }},0,0,0,\frac{{\alpha _h \phi _h }}{{\texttt {k}_1 \texttt {k}_5 - \eta \alpha _h }},0,\frac{{\phi _r }}{{\mu _r }},0,0,0} \right) . \end{aligned}$$

Using the next generation method^33^ we compute the basic reproduction number $\mathcal {R}_{0}$. Let $x = \left( {E_h ,I_h ,C_h ,V_h ,E_r ,I_r } \right)$ then we have

$$\begin{aligned} \frac{{dx}}{{dt}} = \mathcal {F} - \mathcal {V}, \end{aligned}$$

where

$$\begin{aligned} \mathcal {F} = \left( {\begin{array}{*{20}c} {\frac{{\left( {\beta _1 I_h + \beta _2 I_r } \right) S_h }}{{N_h }}} \\ 0 \\ 0 \\ 0 \\ {\frac{{\beta _3 S_r I_r }}{{N_r }}} \\ 0 \\ \end{array}} \right) ,\,\,\,\,\,\mathcal {V} = \left( {\begin{array}{*{20}c} {\texttt {k}_2 E_h } \\ {\texttt {k}_3 I_h - \beta E_h } \\ {\texttt {k}_4 C_h - \gamma I_h } \\ {\texttt {k}_5 V_h - \alpha _h S_h } \\ {\texttt {k}_6 E_r } \\ {\texttt {k}_7 I_r - \varepsilon E_r } \\ \end{array}} \right) . \end{aligned}$$

Then we considered the Jacobian of the Linearized system at disease free state which is given by

$$\begin{aligned} \mathbf{{F}} = \left( {\begin{array}{*{20}c} 0 &{} {\frac{{\beta _1 S_h^0 }}{{N_h^0 }}} &{} 0 &{} 0 &{} 0 &{} {\frac{{\beta _2 S_h^0 }}{{N_h^0 }}} \\ 0 &{} 0 &{} 0 &{} 0 &{} 0 &{} 0 \\ 0 &{} 0 &{} 0 &{} 0 &{} 0 &{} 0 \\ 0 &{} 0 &{} 0 &{} 0 &{} 0 &{} 0 \\ 0 &{} 0 &{} 0 &{} 0 &{} 0 &{} \frac{{\beta _3 S_r^0 }}{{N_r^0 }} \\ 0 &{} 0 &{} 0 &{} 0 &{} 0 &{} 0 \\ \end{array}} \right) ,\,\mathbf{{V}} = \left( {\begin{array}{*{20}c} {\texttt {k}_2 } &{} 0 &{} 0 &{} 0 &{} 0 &{} 0 \\ { - \beta } &{} {\texttt {k}_3 } &{} 0 &{} 0 &{} 0 &{} 0 \\ 0 &{} { - \gamma } &{} {\texttt {k}_4 } &{} 0 &{} 0 &{} 0 \\ 0 &{} 0 &{} 0 &{} {\texttt {k}_5 } &{} 0 &{} 0 \\ 0 &{} 0 &{} 0 &{} 0 &{} {\texttt {k}_6 } &{} 0 \\ 0 &{} 0 &{} 0 &{} 0 &{} { - \varepsilon } &{} {\texttt {k}_7 } \\ \end{array}} \right) . \end{aligned}$$

Where, ${\textbf {F}}$ and ${\textbf {V}}$ are $6\times 6$ matrices calculated as $\mathbf{{F}} = \left( {\frac{{\partial F_i }}{{\partial x_j }}} \right) _{D_0 }$ and $\mathbf{{V}} = \left( {\frac{{\partial V_i }}{{\partial x_j }}} \right) _{D_0 }$. Also, $\mathbf{{F}}$ and $\mathbf{{V}}$ contains the linear and non-linear terms of infected compartments. So, the reproduction number is calculated as

14 $$\begin{aligned} \mathcal {R}_0 = \rho \left( {\mathbf{{FV}}^{ - 1} } \right) = \max \left\{ {\mathcal {R}_r^0 ,\mathcal {R}_h^0 } \right\} = \max \left\{ {\frac{{\varepsilon \beta _3 }}{{\texttt {k}_6 \texttt {k}_7 }},\frac{{\beta \beta _1 S_h^0 }}{{\texttt {k}_2 \texttt {k}_3 N_h^0 }}} \right\} . \end{aligned}$$

### Existence of endemic equilibrium point

The endemic equilibrium (EE) of the fractional monkeypox model is given by

$$ \zeta ^* = \left( {S_h^* ,E_h^* ,I_h^* ,C_h^* ,V_h^* ,S_r^* ,E_r^* ,I_r^* ,R_r^* } \right) ,$$

and is defined as follows

15 $$\begin{aligned} S_h^*&= \frac{{\texttt {k}_5 \phi _h }}{{\left( {\left( {\texttt {k}_1 + \lambda ^{*} _h } \right) \texttt {k}_5 - \eta \alpha _h } \right) }}, \nonumber \\ E_h^*&= \frac{{\lambda ^{*} _h \texttt {k}_5 \phi _h }}{{\texttt {k}_2 \left( {\left( {\texttt {k}_1 + \lambda ^{*} _h } \right) \texttt {k}_5 - \eta \alpha _h } \right) }} = \frac{{\lambda ^{*} _h }}{{\texttt {k}_2 }}S_h^* , \nonumber \\ I_h^*&= \frac{{\beta \lambda ^{*} _h \texttt {k}_5 \phi _h }}{{\texttt {k}_2 \texttt {k}_3 \left( {\left( {\texttt {k}_1 + \lambda ^{*} _h } \right) \texttt {k}_5 - \eta \alpha _h } \right) }} = \frac{{\beta \lambda ^{*} _h }}{{\texttt {k}_2 \texttt {k}_3 }}S_h^* = d_1 \lambda ^{*} _h S_h^*, \nonumber \\ C_h^*&= \frac{{\beta \gamma \lambda ^{*} _h \texttt {k}_5 \phi _h }}{{\texttt {k}_2 \texttt {k}_3 \texttt {k}_4 \left( {\left( {\texttt {k}_1 + \lambda ^{*} _h } \right) \texttt {k}_5 - \eta \alpha _h } \right) }} = \frac{{\beta \gamma \lambda ^{*} _h }}{{\texttt {k}_2 \texttt {k}_3 \texttt {k}_4 }}S_h^* = d_2 \lambda ^{*} _h S_h^* , \nonumber \\ V_h^*&= \frac{{\alpha _h \phi _h }}{{\left( {\left( {\texttt {k}_1 + \lambda ^{*} _h } \right) \texttt {k}_5 - \eta \alpha _h } \right) }} = \frac{\alpha _h S_h^*}{\texttt {k}_{5}}= d_3 \lambda ^{*} _h S_h^*, \nonumber \\ R_h^*&= - \frac{{ - \beta \gamma \rho \lambda ^{*} _h \texttt {k}_5 \phi _h - \beta \texttt {k}_4 \texttt {k}_5 \lambda ^{*} _h \phi _h \omega _1 }}{{\texttt {k}_2 \texttt {k}_3 \texttt {k}_4 \left( {\left( {\texttt {k}_1 + \lambda ^{*} _h } \right) \texttt {k}_5 - \eta \alpha _h } \right) \mu _h }} = d_4 \lambda ^{*} _h S_h^* + d_5 \lambda ^{*} _h S_h^*, \nonumber \\ S_r^*&= \frac{{\phi _r }}{{\left( {\lambda ^{*} _{r} + \mu _r } \right) }}, \nonumber \\ E_r^*&= \frac{{\lambda ^{*} _{r} \phi _r }}{{\texttt {k}_6 \left( {\mu _r + \lambda ^{*} _{r} } \right) }} = \frac{{\lambda ^{*} _r }}{{\texttt {k}_6 }}S_r^* = d_6 \lambda ^{*} _r S_r^* , \nonumber \\ I_r^*&= \frac{{\varepsilon \lambda ^{*} _r \phi _r }}{{\texttt {k}_6 \texttt {k}_7 \left( {\lambda ^{*} _r + \mu _r } \right) }} = \frac{{\lambda ^{*} _r \varepsilon }}{{\texttt {k}_6 \texttt {k}_7 }}S_r^* = d_7 \lambda ^{*} _r S_r^*, \nonumber \\ R_r^*&= \frac{{\varepsilon \lambda ^{*} _r \phi _r \omega _2 }}{{\texttt {k}_6 \texttt {k}_7 \mu _r \left( {\lambda ^{*} _r + \mu _r } \right) }} = \frac{{\lambda ^{*} _r \varepsilon \omega _2 }}{{\texttt {k}_6 \texttt {k}_7 \mu _r }}S_r^* = d_8 \lambda ^{*} _r S_r^*, \nonumber \\ \end{aligned}$$

where, $$d_1 = \frac{\zeta }{{\texttt {k}_3 }},\,d_2 = \frac{{\gamma d_1 }}{{\texttt {k}_4 }},\,d_3 = \frac{{\alpha _h }}{{\texttt {k}_5 }},\,d_4 = \frac{{\rho d_2 }}{{\mu _h }},\,\,d_5 = \frac{{\zeta \omega _1 }}{{\texttt {k}_2 \texttt {k}_3 \mu _h }},\,d_6 = \frac{1}{{\texttt {k}_6 }},\,\,d_7 = \frac{{\varepsilon d_6 }}{{\texttt {k}_7 }},\,d_8 =d_6+d_7\,\,and \,\, d_9=\frac{\beta _{2}}{\beta _{3}d_8}$$.

Moreover,

16 $$\begin{aligned} \,\lambda ^{*} _r&= \frac{{\beta _3 I_{r}^{*} }}{{N_{r}^{*} }} = \frac{{\left( {\mathcal {R}_r^0 - 1} \right) }}{{\left( {d_6 + d_7 + d_8 } \right) }}=\frac{{\left( {\mathcal {R}_r^0 - 1} \right) }}{{ { d_9 }}}, \end{aligned}$$

17 $$\begin{aligned} \lambda ^{*} _h&= \frac{{\beta _1 I_{h}^{*}+\frac{{\beta _2 }}{\beta _3 }\frac{{\left( {\mathcal {R}_r^0 - 1} \right) }}{{d_{9}}}{N_{r}^{*} } }}{{N_{h}^{*} }}, \end{aligned}$$

by using (16) in (17) we have

18 $$\begin{aligned} \lambda ^{*} _h = \frac{{\beta _1 I_{h}^{*} }}{{N_{h}^{*} }} + \frac{{\left( {R_r^0 - 1} \right) {N_{r}^{*} }}}{{d_{9}{N_{h}^{*} } }}\,, \end{aligned}$$

### Global stability at EE

In the system (6) $\lambda _h = \frac{{\beta _1 I_h }}{{N_h }} + \frac{{\beta _2 I_r }}{{N_h }}$ and $\lambda _r = \frac{{\beta _3 I_r }}{{N_r }}$. Also $N_h \le \frac{{\phi _h }}{{\mu _h }},\,\,N_r \le \frac{{\phi _r }}{{\mu _r }}\,as\,t \rightarrow \infty$, therefore, we have $\lambda _{h_1 } = \alpha _3 I_h + \alpha _4 I_r$and $\lambda _{r_1 } = \alpha _5 I_r.$ Therefore, the system (6) becomes

19 $$\begin{aligned} ^c D_t^\vartheta S_h&= \phi _h - \lambda _{h_1 } S_h - \texttt {k}_1 S_h + \eta V_h, \nonumber \\ ^c D_t^\vartheta E_h&= \lambda _{h_1 } S_h - \texttt {k}_2 E_h, \nonumber \\ ^c D_t^\vartheta I_h&= \beta E_h - \texttt {k}_3 I_h,\nonumber \\ ^c D_t^\vartheta C_h&= \gamma I_h - \texttt {k}_4 C_h, \nonumber \\ ^c D_t^\vartheta V_h&= \alpha _h S_h - \texttt {k}_5 V_h ,\,\,\,\,\,\,\,\,\,\,\,\,\,\,\,\,\,\,\,\,\,\,\,\,\,\,\,\,\,\,\,\,\,\,\,\,\,\,\,\,\,\,\,\,\,\,\,\,\,\,\,\,\,\,\,\,\,\,\,\,\,\,\,\,\,\,\,\,\,\,\,\,\,\,\,\,\,\,\,\,\,\,\,\,\,\,\,\,\,\,\,\,\, \nonumber \\ ^c D_t^\vartheta R_h&= \rho C_h + \omega _1 I_h - \mu _h R_h, \nonumber \\ ^c D_t^\vartheta S_r&= \phi _r - \left( {\lambda _{r_1 } + \mu _r } \right) S_r, \nonumber \\ ^c D_t^\vartheta E_r&= \lambda _{r_1 } S_r - \texttt {k}_6 E_r, \nonumber \\ ^c D_t^\vartheta I_r&= \varepsilon E_r - \texttt {k}_7 I_r, \nonumber \\ ^c D_t^\vartheta R_r&= \omega _2 I_r - \mu _r R_r. \end{aligned}$$

Results for system (19) at steady states are calculated as follows

$$\begin{aligned} \phi _h&= \lambda _{_{h_1 } }^* S_h^* + \texttt {k}_1 S_h^* - \eta V_h^* ,\,\,\texttt {k}_2 E_h^* = \lambda _{_{h_1 } }^* S_h^* ,\,\,\texttt {k}_3 I_h^* = \beta E_h^* ,\,\,\texttt {k}_4 C_h^* = \gamma I_h^*,\\ \texttt {k}_5 V_h^*&= \alpha _h S_h^* ,\,\,\phi _r = \left( {\lambda _{r_1 }^* + \mu _r } \right) S_r^* ,\,\,\texttt {k}_6 E_r^* = \lambda _{r_1 }^* S_r^* ,\texttt {k}_7 I_r^* = \,\varepsilon E_r^*. \end{aligned}$$

#### Theorem 4.1

If $\mathcal {R}_{0}>1$, then (19) at EE $\zeta ^{*}$ is global asymptotic stability (GAS) if $\left( {6 - \frac{{S_h ^* }}{{S_h }} + \frac{{\lambda _{h_1 } }}{{\lambda _{h_1 }^* }}\left( {1 - \frac{{S_h E_h^* }}{{S_h ^* E_h }}} \right) - \frac{{I_h^* E_h }}{{I_h E_h^* }} - \frac{{C_h }}{{C_h^* }} - \frac{{C_h^* I_h }}{{C_h I_h^* }} - \frac{{S_r ^* }}{{S_r }} + \frac{{\lambda _{r_1 } }}{{\lambda _{r_1 }^* }}\left( {1 - \frac{{S_r E_r^* }}{{S_r ^* E_r }}} \right) - \frac{{I_r }}{{I_r^* }} - \frac{{I_r^* E_r }}{{I_r E_r^* }}} \right) \le 0.$

#### Proof

Consider the following nonlinear Lyapunov function given by (20)

20 $$\begin{aligned} \mathbf{{M}}\left( t \right)&= \mathbf{{M}}_h \left( t \right) + \mathbf{{M}}_r \left( t \right) , \nonumber \\&= M_1 \left( {S_h - S_h^* - S_h^* \ln \frac{{S_h }}{{S_h^* }}} \right) + M_2 \left( {E_h - E_h^* - E_h^* \ln \frac{{E_h }}{{E_h^* }}} \right) + M_3 \left( {I_h - I_h^* - I_h^* \ln \frac{{I_h }}{{I_h^* }}} \right) \nonumber \\&\quad + M_4 \left( {C_h - C_h^* - C_h^* \ln \frac{{C_h }}{{C_h^* }}} \right) + M_5 \left( {V_h - V_h^* - V_h^* \ln \frac{{V_h }}{{V_h^* }}} \right) + M_6 \left( {S_r - S_r^* - S_r^* \ln \frac{{S_r }}{{S_r^* }}} \right) \nonumber \\&\quad + M_7 \left( {E_r - E_r^* - E_r^* \ln \frac{{E_h }}{{E_r^* }}} \right) + M_8 \left( {I_r - I_r^* - I_r^* \ln \frac{{I_r }}{{I_r^* }}} \right) . \end{aligned}$$

The Caputo fractional derivative of (20) implies

21 $$\begin{aligned} ^c D_t^\vartheta \mathbf{{M}}\left( t \right)&= ^c D_t^\vartheta \mathbf{{M}}_h \left( t \right) + ^c D_t^\vartheta \mathbf{{M}}_r \left( t \right) , \nonumber \\&\le M_1 \left( {1 - \frac{{S_h^* }}{{S_h }}} \right) \,^c D_t^\vartheta S_h \left( t \right) + M_2 \left( {1 - \frac{{E_h^* }}{{E_h }}} \right) \,^c D_t^\vartheta E_h \left( t \right) + M_3 \left( {1 - \frac{{I_h^* }}{{I_h }}} \right) \,^c D_t^\vartheta I_h \left( t \right) \nonumber \\&\quad + M_4 \left( {1 - \frac{{C_h^* }}{{C_h }}} \right) \,^c D_t^\vartheta C_h \left( t \right) + M_5 \left( {1 - \frac{{V_h^* }}{{V_h }}} \right) \,^c D_t^\vartheta V_h \left( t \right) + M_6 \left( {1 - \frac{{S_r^* }}{{S_r }}} \right) \,^c D_t^\vartheta S_r \left( t \right) \nonumber \\&\quad + M_7 \left( {1 - \frac{{E_r^* }}{{E_r }}} \right) \,^c D_t^\vartheta E_r \left( t \right) + M_8 \left( {1 - \frac{{I_r^* }}{{I_r }}} \right) \,^c D_t^\vartheta I_r \left( t \right) , \nonumber \\&= \lambda _{r_{1}}^{*}S_{r}^{*}\Big (\left( {1 - \frac{{S_h^* }}{{S_h }}} \right) \,^c D_t^\vartheta S_h \left( t \right) + \left( {1 - \frac{{E_h^* }}{{E_h }}} \right) \,^c D_t^\vartheta E_h \left( t \right) + \frac{{\texttt {k}_2 }}{\beta }\left( {1 - \frac{{I_h^* }}{{I_h }}} \right) \,^c D_t^\vartheta I_h \left( t \right) + \frac{{\texttt {k}_2 \texttt {k}_3 }}{{\gamma \beta }}\nonumber \\&\quad \left( {1 - \frac{{C_h^* }}{{C_h }}} \right) \,^c D_t^\vartheta C_h \left( t \right) + \frac{{\texttt {k}_1 }}{{\alpha _h }}\left( {1 - \frac{{V_h^* }}{{V_h }}} \right) \,^c D_t^\vartheta V_h \left( t \right) \Big ) +\lambda _{h_{1}}^{*}S_{h}^{*} \Big (\left( {1 - \frac{{S_r^* }}{{S_r }}} \right) \,^c D_t^\vartheta S_r \left( t \right) \nonumber \\&\quad + \left( {1 - \frac{{E_r^* }}{{E_r }}} \right) \,^c D_t^\vartheta E_r \left( t \right) + \frac{{\texttt {k}_6 }}{\varepsilon }\left( {1 - \frac{{I_r^* }}{{I_r }}} \right) \,^c D_t^\vartheta I_r \left( t \right) \Big ), \nonumber \\ \left( {1 - \frac{{S_h^* }}{{S_h }}} \right) \,^c D_t^\vartheta S_h&= \left( {1 - \frac{{S_h^* }}{{S_h }}} \right) \left( {\lambda _{h_1 }^* S_h^* + \texttt {k}_1 S_h^* - \eta V_h^* - \lambda _{h_1 } S_h - \texttt {k}_1 S_h + \eta V_h } \right) , \nonumber \\&= \lambda _{h_{1}}^{*} S_h^* \left( {1 - \frac{{S_h \lambda _{h_{1}} }}{{S_h^* \lambda _{h_{1}}^* }} - \frac{{S_h^* }}{{S_h }} + \frac{{\lambda _{h_{1}} }}{{\lambda _{h_{1}}^* }}} \right) +\texttt {k}_1 S_h^* \left( {2 - \frac{{S_h }}{{S_h^* }} - \frac{{S_h^* }}{{S_h }}} \right) \nonumber \\&\quad - \eta V_h^*\left( 1-\frac{V_{h}}{V_{h}^{*}}\right) \left( 1-\frac{S_{h}^{*}}{S_{h}}\right) , \nonumber \\ \left( {1 - \frac{{E_h^* }}{{E_h }}} \right) \,^c D_t^\vartheta E_h&= \left( {1 - \frac{{E_h^* }}{{E_h }}} \right) \left( {\lambda _{h_1 } S_h - \lambda _{h_1 }^* S_h^* \frac{{E_h }}{{E_h^* }}} \right) , \nonumber \\&= \lambda _{h_{1}}^{*} S_h^* \left( {1 - \frac{{S_h \lambda _{h_{1}} }}{{S_h^* \lambda _{h_{1}}^* }}\frac{{E_h^* }}{{E_h }} - \frac{{E_h }}{{E_h^* }} + \frac{{S_h \lambda _{h_{1}} }}{{S_h^* \lambda _{h_{1}}^* }}} \right) , \nonumber \\ \frac{\texttt {k}_2}{\beta }\left( {1 - \frac{{I_h^* }}{{I_h }}} \right) \,^c D_t^\vartheta I_h &= \frac{\texttt {k}_2}{\beta }\left( {1 - \frac{{I_h^* }}{{I_h }}} \right) \left( {\beta E_h - \texttt {k}_3 \frac{{I_h }}{{I_h^* }}I_h^* } \right) , \nonumber \\&= \lambda _{h_{1}}^{*} S_h^* \left( {1 + \frac{{E_h }}{{E_h^* }} - \frac{{I_h }}{{I_h^* }} - \frac{{E_h I_h^* }}{{E_h^* I_h }}} \right) , \nonumber \\&\quad \frac{\texttt {k}_2\texttt {k}_3}{\gamma \beta }\left( {1 - \frac{{C_h^* }}{{C_h }}} \right) \,^c D_t^\vartheta C_h =\frac{\texttt {k}_2\texttt {k}_3}{\gamma \beta } \left( {1 - \frac{{C_h^* }}{{C_h }}} \right) \left( {\frac{{I_h }}{{I_h^* }} - \frac{{C_h }}{{C_h^* }}} \right) , \nonumber \\&= \lambda _{h_{1}}^{*} S_h^* \left( {1 - \frac{{C_h }}{{C_h^* }} - \frac{{I_h C_h^* }}{{I_h^* C_h }} + \frac{{I_h }}{{I_h^* }}} \right) , \nonumber \\ \frac{\texttt {k}_1}{\alpha _h}\left( {1 - \frac{{V_h^* }}{{V_h }}} \right) \,^c D_t^\vartheta V_h &= \left( {1 - \frac{{V_h^* }}{{V_h }}} \right) \left( {\alpha _h S_h - \texttt {k}_5 V_h^* \frac{{V_h }}{{V_h^* }}} \right) , \nonumber \\&= \texttt {k}_1 S_h^* \left( {1 + \frac{{S_h }}{{S_h^* }} - \frac{{V_h }}{{V_h^* }} - \frac{{V_h^* S_h }}{{V_h S_h^* }}} \right) , \nonumber \\ \left( {1 - \frac{{S_r^* }}{{S_r }}} \right) \,^c D_t^\vartheta S_r&= \left( {1 - \frac{{S_r^* }}{{S_r }}} \right) \left( {\lambda _{r_1 }^* S_r^* + \mu _r S_r^* - \lambda _{r_1 } S_r - \mu _r S_r } \right) , \nonumber \\&= \lambda _{r_{1}}^{*} S_r^* \left( {1 - \frac{{S_r I_r }}{{S_r^* I_r^* }} - \frac{{S_r^* }}{{S_r }} + \frac{{I_r }}{{I_r^* }}} \right) + \mu _r S_r^* \left( {2 - \frac{{S_r }}{{S_r^* }} - \frac{{S_r^* }}{{S_r }}} \right) , \nonumber \\ \left( {1 - \frac{{E_r^* }}{{E_r }}} \right) \,^c D_t^\vartheta E_r & = \left( {1 - \frac{{E_r^* }}{{E_r }}} \right) \left( {\lambda _{r_1 } S_r^* - \lambda _{r_1 }^* S_r^* \frac{{E_r }}{{E_r^* }}} \right) , \nonumber \\&= \lambda _{r_{1}}^{*} S_r^* \left( {1 + \frac{{S_r I_r }}{{S_r^* I_r^* }} - \frac{{E_r }}{{E_r^* }} - \frac{{S_r I_r E_r^* }}{{S_r^* I_r^* E_r }}} \right) , \nonumber \\ \frac{\texttt {k}_6}{\epsilon }\left( {1 - \frac{{I_r^* }}{{I_r }}} \right) \,^c D_t^\vartheta E_r &= \left( {1 - \frac{{I_r^* }}{{I_r }}} \right) \left( {\varepsilon E_r - \varepsilon E_r^* \frac{{I_r }}{{I_r^* }}} \right) , \nonumber \\&= \lambda _{r_{1}}^{*} S_r^* \left( {1 + \frac{{E_r }}{{E_r^* }} - \frac{{I_r^* }}{{I_r }} - \frac{{E_r I_r }}{{E_r^* I_r^* }}} \right) . \end{aligned}$$

After replacement in (21) it implies

$$\begin{aligned}{}&\begin{array}{l} ^c D_t^\vartheta M\left( t \right) \le \lambda _{h_1 }^* S_h^* \lambda _{r_1 }^* S_r^* \left( \begin{array}{l} 6 - \frac{{S_h ^* }}{{S_h }} + \frac{{\lambda _{h_{1}}}}{{\lambda _{h_{1}}^* }}\left( {1 - \frac{{S_h E_h^* }}{{S_h ^* E_h }}} \right) - \frac{{I_h^* E_h }}{{I_h E_h^* }} - \frac{{C_h }}{{C_h^* }} - \frac{{C_h^* I_h }}{{C_h I_h^* }} - \frac{{S_r ^* }}{{S_r }} + \frac{{I _{r } }}{{I _{r }^* }}\left( {1 - \frac{{S_r E_r^* }}{{S_r ^* E_r }}} \right) \\ - \frac{{I_r^* }}{{I_r}} - \frac{{I_r^* E_r }}{{I_r E_r^* }} \\ \end{array} \right) \\ \,\,\,\,\,\,\,\,\,\,\,\,\,\,\,\,\,\,\,\,\,\,\,\,\, - \texttt {k}_1 S_h^* \lambda _{r_1 }^* S_r^* \Big ( {\left( {\frac{{S_h^* }}{{S_h }} - 1 - \ln \frac{{S_h^* }}{{S_h }}} \right) + \left( {\frac{{V_h }}{{V_h^* }} - 1 - \ln \frac{{V_h }}{{V_h^* }}} \right) + \left( {\frac{{S_h V_h^* }}{{S_h^* V_h }} - 1 - \ln \frac{{S_h V_h^* }}{{S_h^* V_h }}} \right) } \Big ) - \eta V_h^* \\ \,\,\,\,\,\,\,\,\,\,\,\,\,\,\,\,\,\,\,\,\,\,\lambda _{r_1 }^* S_r^* \left( {1 - \frac{{V_h }}{{V_h^* }}} \right) \left( {1 - \frac{{S_h^* }}{{S_h }}} \right) - \mu _r \lambda _{h_1 }^* S_h^* S_r^* \Big (\frac{{\left( {S_r - S_r^* } \right) ^2 }}{{S_r S_r^* }}\Big ), \\ \end{array} \\&\begin{array}{l} ^c D_t^\vartheta M\left( t \right) \le \lambda _{h_1 }^* S_h^* \frac{{\alpha _5 \left( {\mathcal {R}_r^0 - 1} \right) \phi _r }}{{\beta _3 c_9 \mu _r }}S_r^* \left( \begin{array}{l} 6 - \frac{{S_h ^* }}{{S_h }} + \frac{{\lambda _{h_1 } }}{{\lambda _{h_1 }^* }}\left( {1 - \frac{{S_h E_h^* }}{{S_h ^* E_h }}} \right) - \frac{{I_h^* E_h }}{{I_h E_h^* }} - \frac{{C_h }}{{C_h^* }} - \frac{{C_h^* I_h }}{{C_h I_h^* }} - \frac{{S_r ^* }}{{S_r }} + \frac{{\lambda _{r_1 } }}{{\lambda _{r_1 }^* }} \\ \left( {1 - \frac{{S_r E_r^* }}{{S_r ^* E_r }}} \right) - \frac{{I_r }}{{I_r^* }} - \frac{{I_r^* E_r }}{{I_r E_r^* }} \\ \end{array} \right) \\ \,\,\,\,\,\,\,\,\,\,\,\,\,\,\,\,\,\,\,\,\,\,\,\,\, - \alpha _5 \frac{{\left( {R_r^0 - 1} \right) \phi _r }}{{\beta _3 c_9 \mu _r }}S_r^* \left( \begin{array}{l} \texttt {k}_1 S_h^* \left( {\left( {\frac{{S_h^* }}{{S_h }} - 1 - \ln \frac{{S_h^* }}{{S_h }}} \right) + \left( {\frac{{V_h }}{{V_h^* }} - 1 - \ln \frac{{V_h }}{{V_h^* }}} \right) + \left( {\frac{{S_h V_h^* }}{{S_h^* V_h }} - 1 - \ln \frac{{S_h V_h^* }}{{S_h^* V_h }}} \right) } \right) \\ + \eta V_h^* \left( {1 - \frac{{V_h }}{{V_h^* }}} \right) \left( {1 - \frac{{S_h^* }}{{S_h }}} \right) \\ \end{array} \right) \\ \,\,\,\,\,\,\,\,\,\,\,\,\,\,\,\,\,\,\,\,\,\, - \mu _r \lambda _{h_1 }^* S_h^* S_r^* \frac{{\left( {S_r - S_r^* } \right) ^2 }}{{S_r S_r^* }}. \end{array} \end{aligned}$$

Thus, $$^c D_t^\vartheta \mathbf{{M}}\left( t \right) \le 0$$ for $$\mathcal {R}_{0}>1.$$ Furthermore, $$^c D_t^\vartheta \mathbf{{M}}\left( t \right) = 0$$ whenever $$\zeta ^* = \left( {S_h^* ,E_h^* ,I_h^* ,C_h^* ,V_h^* ,R_h^* ,S_r^* ,E_r^* ,I_r^* 
} \right) $$. Thus, by LaSalle’s invariance principle the *EE* is *GAS* in $$\Omega $$ whenever, $$\mathcal {R}_{0}>1.$$ $$\square $$

## Interpretations of $\mathcal {R}_0$ versus model parameters

**Figure 2:**
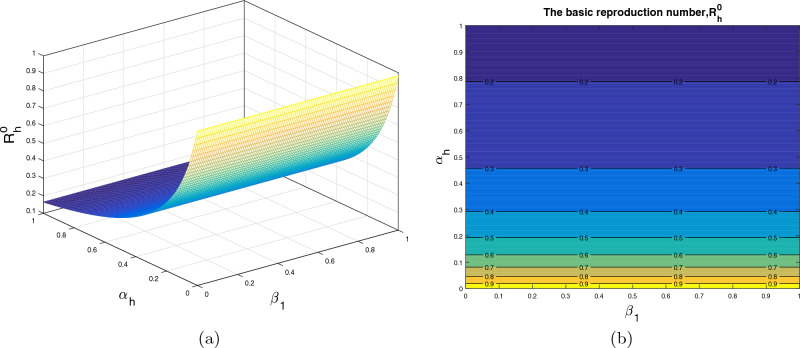
The subplot (**a**) illustrate the impact of $\beta _1$ (disease transmission coefficient relative to $I_h$) and $\alpha _h$ (vaccination rate ) on $\mathcal{{R}}_{0}$, where (**b**) shows the respective contour plot.

**Figure 3:**
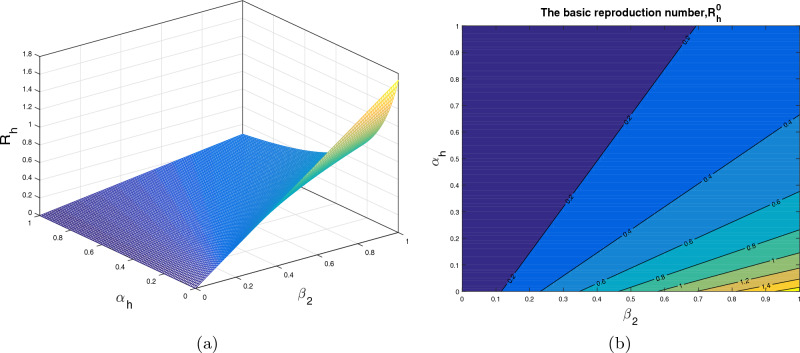
The subplot (**a**) analyze the impact of $\beta _2$ (disease transmission coefficient relative to $I_r$) and $\alpha _h$ (vaccination rate ) on $\mathcal{{R}}_{0}$, where (**b**) shows the respective contour plot.

**Figure 4:**
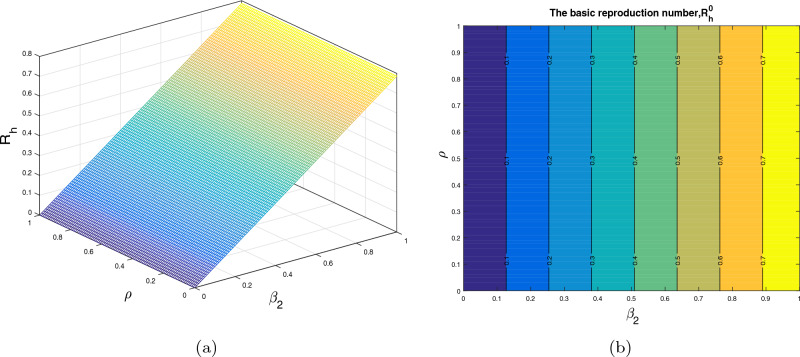
The subplot (**a**) demonstrate the impact of $\beta _2$ (disease transmission coefficient relative to $I_r$) and $\rho$ (recovery rate of $C_h$) on $\mathcal{{R}}_{0}$, where (**b**) shows the respective contour plot.

**Figure 5:**
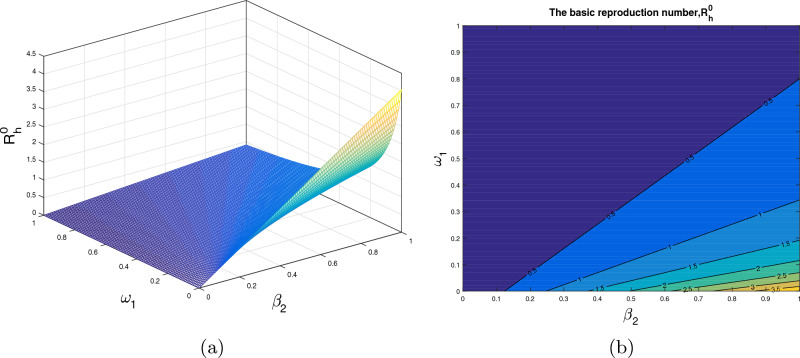
The subplot (**a**) demonstrate the impact of $\beta _2$ (disease transmission coefficient relative to $I_r$) and $\omega _1$ (recovery rate of $I_h$) on $\mathcal{{R}}_{0}$, where (**b**) shows the respective contour plot.

**Figure 6:**
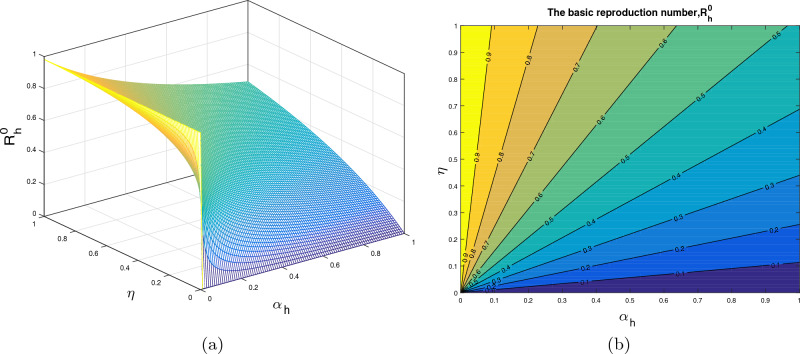
The subplot (**a**) demonstrate the impact of $\alpha _h$ (vaccination rate) and $\eta$ (loss of immunity rate) on $\mathcal{{R}}_{0}$, where (**b**) shows the respective contour plot.

In this section, we analyze the impact of some model parameters on $\mathcal {R}_0$. The basic reproduction number $\mathcal {R}_0$ is a key biological parameter that plays an important role in describing the disease dynamics and its possible control. In these graphical results, we consider the disease transmission rates $\beta _1$, $\beta _2$, vaccination rate $\alpha _h$, recovery rates $\rho ,$ $\omega _1$ and vaccine waning rate $\eta$. The respective graphical results are shown in Figs. 2a, 3, 4, 5 and 6a, while the corresponding contour plots in each case are shown in Figs. 2b, 3, 4, 5 and 6b. Figure 2 shows the combine impact of $\beta _1$ and $\alpha _h$ on $\mathcal {R}_0$. It shows that the $\mathcal {R}_0$ decreases with a decrease in transmission coefficient $\beta _1$ and an increase in vaccination rate $\alpha _h$. Further, impact of $\beta _2$ along with $\alpha _h$ upon $\mathcal {R}_0$ is demonstrated in the Fig. 3a with the corresponding counter plot 3b. It can be observed that with the increase in vaccination rate and reduction in contact rate $\beta$ the basic reproduction reduces to a value less than 1. Similarly, the impact of $\rho$ and $\beta _2$, $\omega _1$ and $\beta _2$, $\eta$ and $\alpha _h$ are presented in Figs. 4a, 5a, and 6a respectively. We can observe that with the decrease in disease transmission rate $\beta _2$ and enhancement in vaccination rate $\alpha _h$ and recovery rate $\omega$ the basic reproduction decreases significantly to a value less than unity.

## Numerical results of the fractional model

In this section, we first investigate the numerical solution of the Caputo fractional monkeypox model. The generalized fractional Adams-Bash forth-Moulton approach^34^, an effective iterative method is used to solve the model numerically. The simulation for different model parameters and different values of $\vartheta$ are carried out using the generated numerical technique and the values for model parameters are listed in Table 1. The subsequent subsection contains the iterative solution.

### Numerical technique

A concise numerical method for the approximate solution of the monkeypox transmission model in the Caputo sense is shown in this subsection. For this purpose, the fractional Adams-Bashforth-Moulton is used. The system (6) can be recomposed in the subsequent problem to get the desired scheme:

22 $$\begin{aligned} \left\{ \begin{array}{ll} {}^{{C}} D^{\vartheta }_{t} {\textbf {v}}(t)=\mathcal {M}\Big (t,{\textbf {v}}(t)\Big ),\;\; 0<t<\mathcal {T},\\ {\textbf {v}}^{(m)}(0)={\textbf {v}}^{(m)}_0,~~~m=0,1,\ldots ,v,~~v=[\vartheta ], \end{array} \right. \end{aligned}$$

where, ${\textbf {v}}=(S_{h},E_{h},I_{h},C_h,V_{h},R_{h},S_{r},E_{r},I_{r},R_{r})\in \mathbb {R}_{+}^{10}$, and $\mathcal {M}\big (t,{\textbf {v}}(t)\big )$ shows a continuous real valued function. Using the concept of integral in Caputo case, the aforementioned Eq. (22) is considered as follows:

23 $$\begin{aligned} {\textbf {v}}(t)=\sum ^{~~v-1}_{\texttt {m}=0} {\textbf {v}}^{(m)}_0\frac{t^{m}}{m !}+\frac{1}{\Gamma (\vartheta )}\int _0^t (t-x)^{\vartheta -1}\mathcal {M}\Big (x,{\textbf {v}}(x)\Big )dx. \end{aligned}$$

In order to use the procedure in^34^, a uniform grid on $[0, \mathcal {T}]$ with step size $h = \frac{\mathcal {T}}{N}$, $N\in \mathbb {N}$, where $t_u=uh,~~u=0,1\ldots ,N$ is considered. Thus, the proposed model in (6) can be treated as:

$$\begin{aligned} S_{h_{u+1}}(t)= & {} S_{h_{0}}+\frac{h^{\vartheta }}{\Gamma {\left( \vartheta +2 \right) }} \Big \{\Phi _{h}- \left( \alpha _{1}I_{h}^{m}+\alpha _{2}I_{r}^{m}\right) \frac{S_{h}^{m}}{N_{h}^{m}}-\texttt {k}_{1} S_{h}^{m}+\eta V_{h}^{m}\Big \}+\\\\{} & {} \frac{h^{\vartheta }}{\Gamma {(\vartheta +2)}}\sum _{k=0}^u b_{k,u+1}\Big \{\Phi _{h}- \left( \alpha _{1}I_{h_{k}}+\alpha _{2}I_{r_{k}}\right) \frac{S_{h_{k}}}{N_{h_{k}}}-\texttt {k}_{1} S_{h_{k}}+\eta V_{h_{k}}\Big \},\\\\E_{h_{u+1}}(t)= & {} E_{h_{0}}+\frac{h^{\vartheta }}{\Gamma {(\vartheta +2)}} \Big \{(\alpha _{1}I_{h}^{m}+\alpha _{2}I_{r}^{m})\frac{S_{h}^{m}}{N_{h}^{m}}+\texttt {k}_{2}E_{h}^{m}\Big \}+\frac{h^{\vartheta }}{\Gamma {(\vartheta +2)}}\\\\{} & {} \sum _{j=0}^u b_{k,u+1}\Big \{(\alpha _{1}I_{h_{k}}+\alpha _{2}I_{r_{k}})\frac{S_{h_{k}}}{N_{h_{k}}}+\texttt {k}_{2}E_{h_{k}}\Big \},\\\\I_{h_{u+1}}(t)= & {} I_{h_{0}}+\frac{h^{\vartheta }}{\Gamma {(\vartheta +2)}} \Big \{ \beta E_{h}^{m}-\texttt {k}_{3}I_{h}^{m}\Big \}+\frac{h^{\vartheta }}{\Gamma {(\vartheta +2)}}\sum _{k=0}^u b_{k,u+1}\Big \{E_{h_{k}}-\texttt {k}_{3}I_{h_{k}}\Big \},\\\\C_{h_{u+1}}(t)= & {} C_{h_{0}}+\frac{h^{\vartheta }}{\Gamma {(\vartheta +2)}} \Big \{ \gamma I_{h}^{m}-\texttt {k}_{4}C_{h}^{m}\Big \}+\frac{h^{\vartheta }}{\Gamma {(\vartheta +2)}}\sum _{k=0}^u b_{k,u+1}\Big \{\gamma I_{h_{k}}-\texttt {k}_{4}C_{h_{k}}\Big \},\\\\V_{{h}_{u+1}}(t)= & {} V_{h_{0}}+\frac{h^{\vartheta }}{\Gamma {(\vartheta +2)}} \Big \{\alpha _{h}S_{h}^{m}-\texttt {k}_{5} V_{h}^{m} \Big \}+\frac{h^{\vartheta }}{\Gamma {(\vartheta +2)}}\sum _{k=0}^u b_{k,u+1}\Big \{\alpha _{h}S_{h_{k}}-\texttt {k}_{5} V_{h_{k}} \Big \},\\\\R_{h_{k+1}}(t)= & {} R_{h_{0}}+\frac{h^{\vartheta }}{\Gamma {\left( \vartheta +2 \right) }} \Big \{\rho C_{h}^{m}+\omega _{1} I_{h}^{m}-\mu _{h} R_{h}^{m}\Big \}+\frac{h^{\vartheta }}{\Gamma {(\vartheta +2)}}\sum _{k=0}^u b_{k,u+1}\Big \{\rho C_{h_{k}}+\omega _{1} I_{h_{k}}-\mu _{h} R_{h_{k}} \Big \},\\\\S_{r_{u+1}}(t)= & {} S_{r_{0}}+\frac{h^{\vartheta }}{\Gamma {(\vartheta +2)}} \Big \{\phi _{r}-\frac{\beta _3 I_{r}^{m}}{N_{r}^{m}}S_{r}^{m}-\mu _{r}S_{r}^{m}\Big \}+\frac{h^{\vartheta }}{\Gamma {(\vartheta +2)}}\sum _{k=0}^u b_{k,u+1}\Big \{\phi _{r}-\frac{\beta _3 I_{r_{k}}}{N_{r_{k}}}S_{r_{k}}-\mu _{r}S_{r_{k}} \Big \},\\\\E_{r_{u+1}}(t)= & {} E_{r_{0}}+\frac{h^{\vartheta }}{\Gamma {(\vartheta +2)}} \Big \{\frac{\beta _3 I_{r}^{m}}{N_{r}^{m}}S_{r}^{m}-\texttt {k}_{6}E_{r}^{m}\Big \}+\frac{h^{\vartheta }}{\Gamma {(\vartheta +2)}}\sum _{k=0}^u b_{k,u+1}\Big \{\frac{\beta _3 I_{r_{k}}}{N_{r_{k}}}S_{r_{k}}-\texttt {k}_{6}E_{r_{k}}\Big \},\\\\I_{r_{u+1}}(t)= & {} I_{r_{0}}+\frac{h^{\vartheta }}{\Gamma {(\vartheta +2)}} \Big \{\epsilon E_{r}^{m}-\texttt {k}_{7}I_{r}^{m}\Big \}+\frac{h^{\vartheta }}{\Gamma {(\vartheta +2)}}\sum _{k=0}^u b_{k,u+1}\Big \{\epsilon E_{r_{j}}-\texttt {k}_{7}I_{r_{j}}\Big \},\\\\R_{r_{k+1}}(t)= & {} R_{r_{0}}+\frac{h^{\vartheta }}{\Gamma {(\vartheta +2)}} \Big \{\omega _{2} I_{r}^{m}-\mu _{r}R_{r}^{m}\Big \}+\frac{h^{\vartheta }}{\Gamma {(\vartheta +2)}}\sum _{k=0}^u b_{i,u+1}\Big \{\omega _{2} I_{r_{k}}-\mu _{r}R_{r_{k}}\Big \}, \end{aligned}$$

where,

$$\begin{aligned} S^{m}_{h_{u+1}}(t)= & {} S_{h_{0}}+\frac{1}{\Gamma {(\vartheta )}}\sum _{k=0}^u \theta _{k,u+1}\Big \{\Phi _{h}- \left( \alpha _{1}I_{h_{k}}+\alpha _{2}I_{r_{k}}\right) \frac{S_{h_{k}}}{N_{h_{k}}}-\texttt {k}_{1} S_{h_{k}}+\eta V_{h_{k}}\Big \},\\\\E^{m}_{h_{u+1}}(t)= & {} E_{h_{0}}+\frac{1}{\Gamma {(\vartheta )}}\sum _{k=0}^u \theta _{k,u+1}\Big \{ \left( \alpha _{1}I_{h_{k}}+\alpha _{2}I_{r_{k}}\right) \frac{S_{h_{k}}}{N_{h_{k}}}-\texttt {k}_{2} E_{h_{k}}\Big \},\\\\I^{m}_{h_{u+1}}(t)= & {} I_{h_{0}}+\frac{1}{\Gamma {(\vartheta )}}\sum _{k=0}^u \theta _{k,u+1}\Big \{ \beta E_{h_{k}}-\texttt {k}_{3}I_{h_{k}}\Big \},\\\\C^{m}_{h_{u+1}}(t)= & {} C_{h_{0}}+\frac{1}{\Gamma {(\vartheta )}}\sum _{k=0}^u \theta _{k,u+1}\Big \{\rho C_{h_{k}}+\omega _{1} I_{h_{k}}-\mu _{h} R_{h_{k}} \Big \},\\\\V^{m}_{h_{u+1}}(t)= & {} V_{h_{0}}+\frac{1}{\Gamma {(\vartheta )}}\sum _{k=0}^u \theta _{k,u+1}\Big \{\alpha _{h}S_{h_{k}}-\texttt {k}_{5} V_{h_{k}} \Big \},\\\\R^{m}_{h_{u+1}}(t)= & {} R_{h_{0}}+\frac{1}{\Gamma {(\vartheta )}}\sum _{k=0}^u \theta _{k,u+1}\Big \{\rho C_{h_{k}}+\omega _{1} I_{h_{k}}-\mu _{h} R_{h_{k}}\Big \},\\\\S^{m}_{r_{u+1}}(t)= & {} S_{r_{0}}+\frac{1}{\Gamma {(\vartheta )}}\sum _{k=0}^u \theta _{k,u+1}\Big \{\phi _{r}-\frac{\beta _3 I_{r_{k}}}{N_{r_{k}}}S_{r_{k}}-\mu _{r}S_{r_{k}}\Big \},\\\\E^{m}_{r_{u+1}}(t)= & {} E_{r_{0}}+\frac{1}{\Gamma {(\vartheta )}}\sum _{k=0}^u \theta _{k,u+1}\Big \{\frac{\beta _3 I_{r_{k}}}{N_{r_{k}}}S_{r_{k}}-\texttt {k}_{6}E_{r_{k}}\Big \},\\\\I^{m}_{r_{k+1}}(t)= & {} I_{r_{0}}+\frac{1}{\Gamma {(\vartheta )}}\sum _{k=0}^u \theta _{k,u+1}\Big \{\epsilon E_{r_{k}}-\texttt {k}_{7}I_{r_{k}}\Big \},\\\\R^{m}_{r_{u+1}}(t)= & {} R_{r_{0}}+\frac{1}{\Gamma {(\vartheta )}}\sum _{k=0}^u \theta _{k,u+1}\Big \{\omega _{2} I_{r_{k}}-\mu _{r}R_{r_{k}}\Big \},\\\\\end{aligned}$$

Further, we have in the above expressions

$$\begin{aligned} b_{k,u+1}= {\left\{ \begin{array}{ll} u^{\vartheta +1}-(u-\vartheta )(u+1)^{\vartheta },~~~k=0\\ (u-k+2)^{\vartheta +1}+(u-k)^{\vartheta +1}-2(u-k+1)^{\vartheta +1},~~~1\le k \le u,\\ 1,~~~k=u+1, \end{array}\right. } \end{aligned}$$

and

$$\begin{aligned} \theta _{k,u+1}=\frac{h^{\vartheta }}{\vartheta }\left[ (u-k+1)^{\vartheta }+(u-k)^{\vartheta }\right] ,~~~0\le k \le u. \end{aligned}$$

### Simulation of the fractional monkeypox model

In this section, we applied the iterative technique developed in the preceding section in order to simulate the monkeypox Caputo epidemic model (6). The simulation results are illustrated by taking the baseline values of estimated parameters listed in Table 1. In the simulation results, we mainly focus on the dynamics of infected human and animal populations. Initially, in the simulation, we analyze the dynamics of model state variables for various values of $\vartheta \in (0,1]$ to study the impact of memory index on disease incidence. Further, the influence of variation in the recovery rate $\omega _{1}$ on the behavior of the infected human population is predicted for many values of fractional order $\vartheta$. Moreover, we considered different scenarios by decreasing the effective contact rates among susceptible humans and infected animals (i.e., $\beta _1,\beta _2$) and the parameter $\beta _3$ showing the effective contact rate between susceptible and infected animals. In addition, these simulations are performed for various non-integer values of the parameter $\vartheta$. The detailed discussion of the aforementioned simulation is described in the following subparts.

#### Impact of memory index ($\vartheta$)

**Figure 7:**
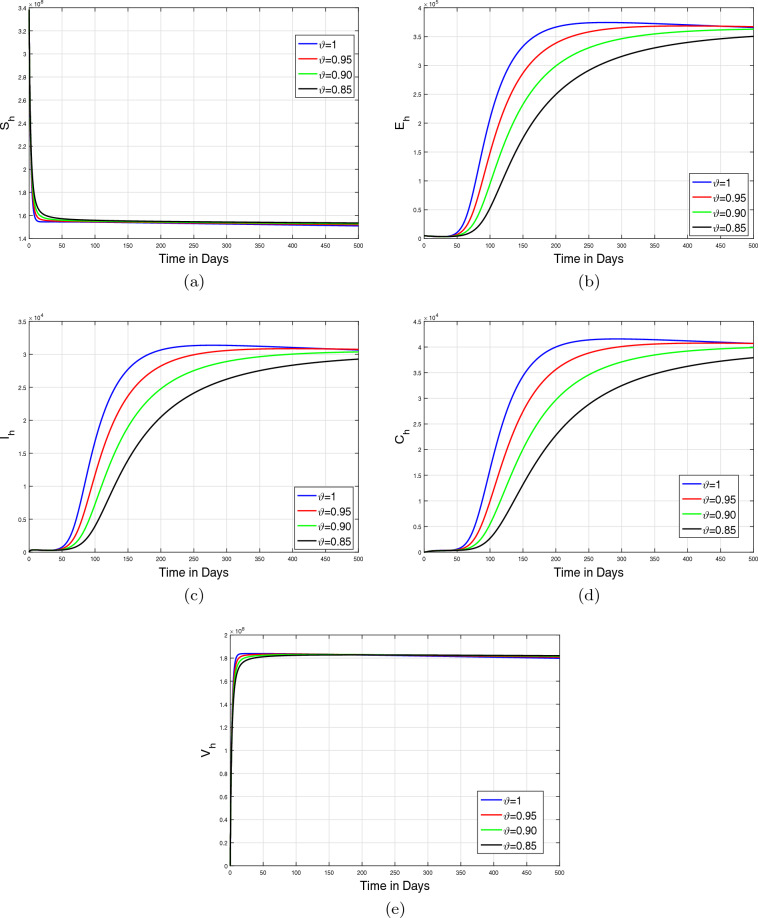
Simulations of only human classes in fractional monkeypox epidemic model (6) for various values of fractional order $\vartheta$.

**Figure 8:**
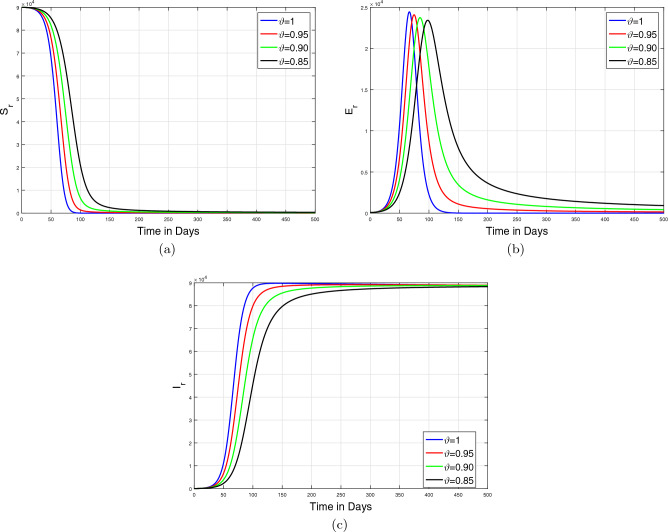
Simulations of only non-human classes in fractional monkeypox epidemic model (6) for various values of fractional order $\vartheta$.

In the first case of simulation, we illustrate the impact of only fractional order $\vartheta$ of the Caputo operator on the model dynamics. The graphical results are shown in Figs. 7a–e and 8a–c. The dynamics of only human population classes are shown in Fig. 7 while Fig. 8 illustrates the dynamical aspects of only animal subpopulation. The simulation in both cases is performed for four values of $\vartheta =1,0.95,0.90,0.85$. The time length considered in the simulation is taken to 500 days. It is observed that under the specific conditions, the solution trajectories in both population classes are converged to the steady state for all values of fractional order $\vartheta$.

#### Impact of recovery rate $\omega _1$ and memory index ($\vartheta$)

**Figure 9:**
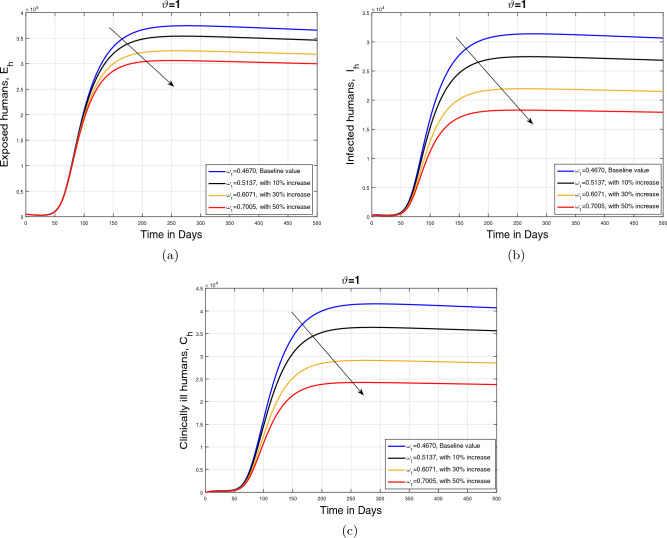
Impact of recovery rate $\omega _1$ with $\vartheta =1$.

**Figure 10:**
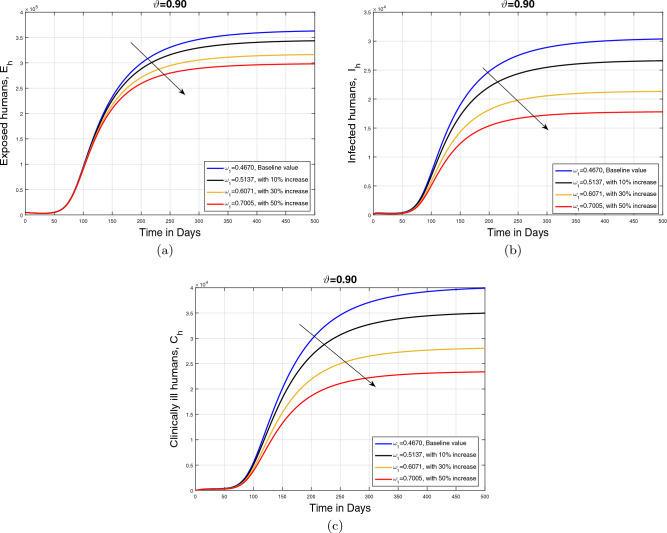
Impact of recovery rate $\omega _1$ with $\vartheta =0.90$.

**Figure 11:**
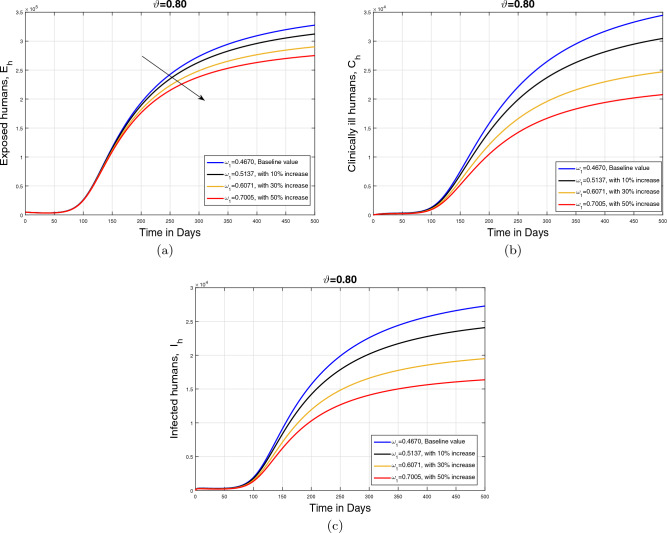
Impact of recovery rate $\omega _1$ with $\vartheta =0.80$.

In this subpart, we analyzed the impact of variation in recovery rate $\omega _1$ on the dynamics of cumulative cases of exposed $E_{h}$, infected $I_{h}$, and clinically infected $C_{h}$ human under different values of memory index ($\vartheta$). The parameter showing recovery rate is perturbed with different rates (with baseline and with $10\%$, $30\%$, and $50\%$ increase) depending on the treatment strength provided to the infected human population. The resulting simulation is accomplished in Figs. 9, 10 and 11 with subplots (a–c) for three values of fractional order $\vartheta$. It is observed that with an increase in parameter $\omega _1$, the infected population in all aforementioned classes reduces showing the impact of treatment on the disease incidence. Additionally, it can observed that in the case of smaller values of $\vartheta$, the decline in the infected population is slightly faster.

#### Impact of disease transmission rates and memory index ($\vartheta$)

**Figure 12:**
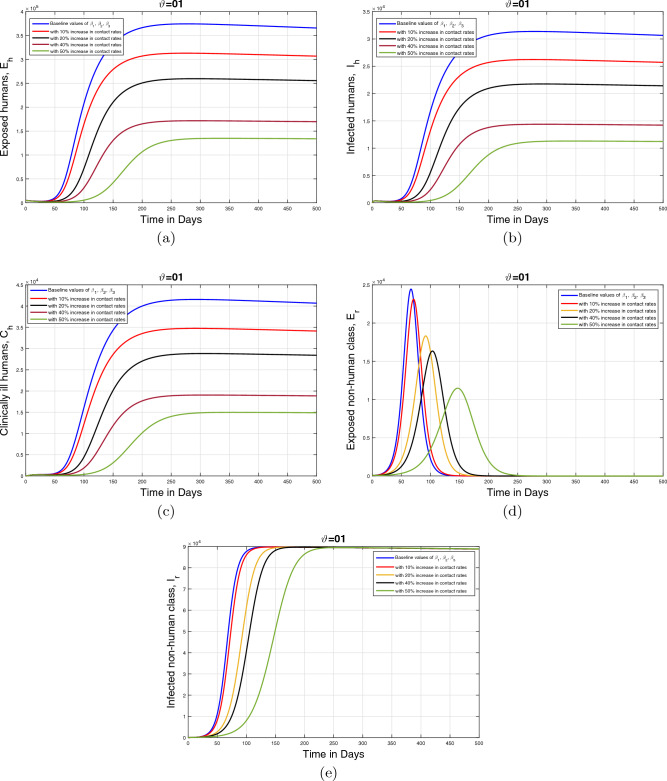
Impact of contact rates $\beta _1, \;\beta _{2}, \;\beta _{3}$ with different rated and $\vartheta =1$.

**Figure 13:**
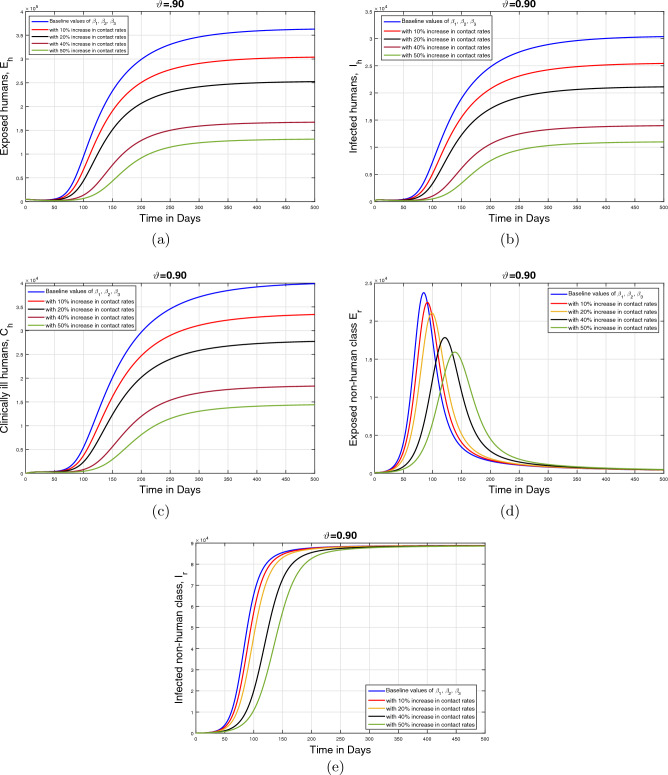
Impact of contact rates$\beta _1, \;\beta _{2}, \;\beta _{3}$ with different rated and $\vartheta =0.90$.

**Figure 14:**
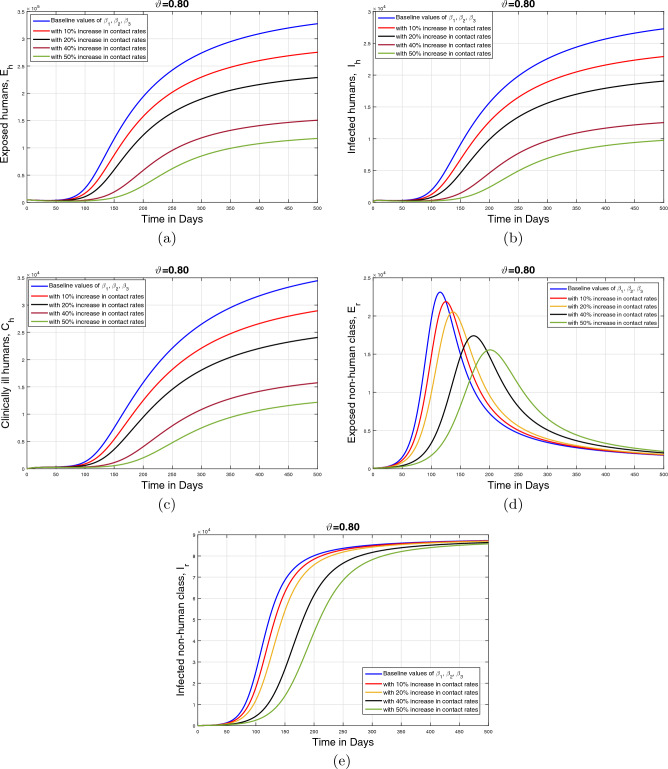
Impact of contact rates $\beta _1, \;\beta _{2}, \;\beta _{3}$ with different rated and $\vartheta =0.80$.

This section describes the influence of the infection transmission rates $\beta _1, \beta _2$, and $\beta _3$ on the dynamics of infected human and animal population classes. The graphical results are initially performed for the baseline values of the aforementioned parameters given in Table 1. Subsequently, the parameter values are decreased with $10\%,$ $20\%,$ $40\%$, and $50\%$ to the estimated values. Furthermore, the simulation results are presented for three values of fractional order $\vartheta$. These resulting simulations for the human population are shown in Figs. 12, 13 and 14 with respective subplots from (a–c) while the dynamics of the infected animal population are demonstrated in Figs. 12, 13 and 14d, e. In all plots, it is evident that the cumulative infected population significantly decreases as the infection transmission rates are reduced. Moreover, it can be seen from Figs. 13 and 14, that fractional values of $\vartheta$ provide more insights enhancing our understanding of the disease dynamics. disease dynamics. Consequently, these graphical interpretations suggest that the effective control of the monkeypox disease and the reduction of disease-induced mortality can be achieved by implementing appropriate treatment and disinfection techniques.

## Conclusion

In this manuscript, we analyzed the dynamics of a novel monkeypox infection with the case study of the recently reported outbreak. The model is first formulated using an integer-order nonlinear system of ten differential equations. The human population was divided into six distinct subgroups, while the animal population was divided into four classes. To estimate the model parameters, we fit the model to the actual cases of the 2022 monkeypox outbreak in the USA. Moreover, keeping the importance of the fractional modeling approach, the integer case model is extended to fractional order via the well-known Caputo operator. In the initial stage, we presented a comprehensive theoretical analysis of the fractional monkeypox model, including the existence and uniqueness of the solutions. The existence and stability analysis of the model equilibria are provided. Furthermore, the fractional model is solved numerically using the fractional Adams-Bashforth-Moulton approach. The simulation results illustrate the impact of disease incidence graphically, considering both the baseline values of the parameters and different values of the fractional order of the Caputo operator. Moreover, the model is simulated by increasing the parameter $\omega _1$ (the recovery rate) and decreasing the disease transmission coefficients $\beta _1, \beta _2, \beta _3$ with different rates to their baseline values. The implementation of a real-data set of monkeypox infections makes this study more visible and important within the existing literature.

## Data Availability

The data that support the findings of this study are available from the corresponding author up on reasonable request. Further, no experiments on humans and/or the use of human tissue samples involved in this study.
